# Development of a Sensitive Screening Method for Simultaneous Determination of Nine Genotoxic Nitrosamines in Active Pharmaceutical Ingredients by GC-MS

**DOI:** 10.3390/ijms232012125

**Published:** 2022-10-12

**Authors:** Anna B. Witkowska, Joanna Giebułtowicz, Magdalena Dąbrowska, Elżbieta U. Stolarczyk

**Affiliations:** 1 Research Analytics Team, Analytical Department, Łukasiewicz Research Network—Industrial Chemistry Institute, 8 Rydygiera Street, 01-793 Warsaw, Poland; 2 Department of Bioanalysis and Drugs Analysis, Doctoral School, Medical University of Warsaw, 61 Żwirki i Wigury, 02-091 Warsaw, Poland; 3 Department of Bioanalysis and Drugs Analysis, Faculty of Pharmacy, Medical University of Warsaw, 1 Banacha, 02-097 Warsaw, Poland; 4 Spectrometric Methods Department, National Medicines Institute, Chełmska 30/34, 00-725 Warsaw, Poland

**Keywords:** nitrosamines, gas chromatography–mass spectrometry, active pharmaceutical ingredient, ionization, microextraction, validation

## Abstract

A worldwide crisis with nitrosamine contamination in medical products began in 2018. Therefore, trace-level analysis of nitrosamines is becoming an emerging topic of interest in the field of quality control. A novel GC-MS method with electron ionization and microextraction was developed and validated for simultaneous determination of nine carcinogenic nitrosamines (NDMA, NMEA, NDEA, NDBA, NMOR, NPYR, NPIP, NDPA, and *N*-methyl-npz) in active pharmaceutical ingredients (APIs): cilostazol, sunitinib malate, and olmesartan medoxomil. The method was validated according to the International Council for Harmonisation of Technical Requirements for Pharmaceuticals for Human Use (ICH) guidelines, demonstrating good linearity in the range of LOQ up to 21.6 ng/mL (120% of specification limit). The limits of detection for the nine nitrosamines were determined to be in the range 0.15–1.00 ng/mL. The developed trace level GC-MS method turned out to be specific, accurate, and precise. The accuracy of all the tested APIs ranged from 94.09% to 111.22% and the precision evaluated by repeatability, intermediate precision, and system precision was RSD ≤ 7.65%. Nitrosamines were not detected in cilostazol and sunitinib, whereas in olmesartan medoxomil NDEA was detected at the level of LOQ. The novel protocol was successfully applied for nitrosamines determination in selected APIs and can be used for the routine quality control of APIs under Good Manufacturing Practices rules, ensuring the safety and effectiveness of pharmaceutical products.

## 1. Introduction

**Figure 1:**
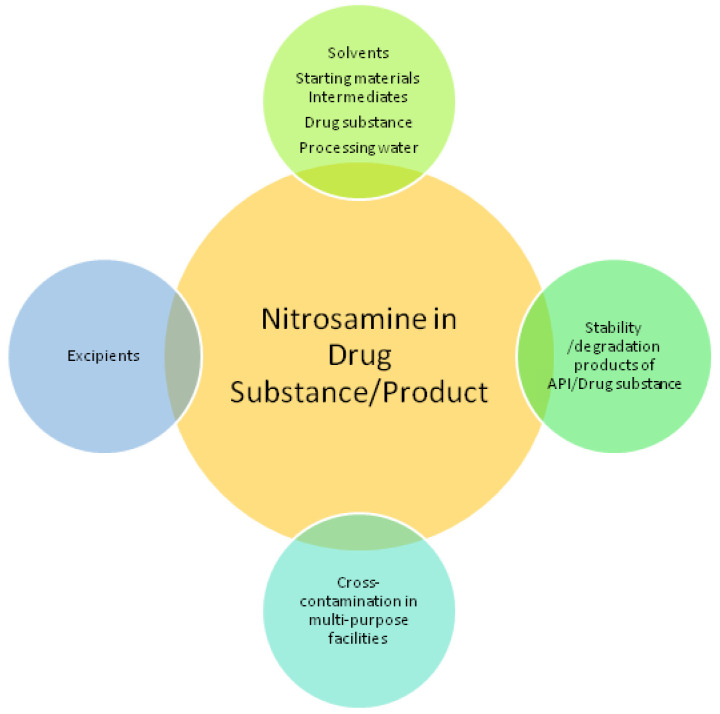
Root causes of nitrosamine impurities in APIs and drug products.

A worldwide crisis with nitrosamine contamination in medical products began in 2018 [1]. Since then, widespread investigations by regulatory agencies, including the European Medicines Agency (EMA) and United States Food and Drug Administration (US FDA), were undertaken. In drugs such as angiotensin II receptor blockers (ARBs), ranitidine, metformin, rifampin, rifapentine, and, recently, verenicline, *N*-nitrosodimethylamine (NDMA) and other nitrosamines have been detected [2,3]. Nitrosamines are known as probable human carcinogens (IARC 2A group for NDMA), potent genotoxic agents, and “cohort of concern” compounds according to ICH M7 guidance [4,5]. They can form in reaction of amine source (e.g., 2°, 3° amines_,_ amidines, hydrazines) and nitrosating agents (e.g., nitrite in acidic conditions, nitrogen oxide, nitrous acid). According to newly established guidelines, every marketing authorization holder has to identify the risk of nitrosamine formation or cross-contamination in the manufacturing/storage of chemical and biological medicines. Root causes of nitrosamine impurities in APIs (active pharmaceutical ingredients) and drug products can be manufacturing process related as well as stability of the drug substance/product or excipient compatibility related (Figure 1) [6,7,8].

The scale of the problem is revealed by the fact that, till now, more than one thousand batches of medical products have been recalled from the market due to the detection of harmful nitrosamines [9].

A large number of studies also resulted in the introduction of new, more restrictive legislation that regulated the concentration of nitrosamines in drug products. Therefore, trace-level analysis of nitrosamines is becoming an emerging topic of interest in the field of quality control. Till now, a wide range of quantitative analytical methods to measure nitrosamine traces in pharmaceuticals have been established, including gas chromatography with single quadrupole or tandem mass spectrometers (GC-MS/MS) with a headspace system or direct injection [10,11,12,13,14,15,16], liquid chromatography–tandem mass spectrometers (LC-MS/MS) or high-resolution mass spectrometers [17,18,19,20], and liquid chromatography with UV detectors and supercritical fluid chromatography (SFC) [21,22,23]. Solid-phase extraction (SPE), solid-phase microextraction (SPME), and dispersive liquid–liquid microextraction (DLLME) are also commonly used with GC-MS to produce comparable results of the LOQ (range 0.5–1.5 ppb) to LC-MS/MS [24,25,26]. Many already established methods are complex and can be applied to only one drug product/API, such as ranitidine and metformin. Our newly established, sensitive analytical screening procedure involving microextraction is fast, simple, environmentally friendly, and with limits of quantitation (LOQs) close to multi-step methods involving SPME or SPE extraction. Moreover GC-MS offers low operational costs, and is commonly used for analysis of volatile impurities in APIs. We demonstrate, for the first time, that our analytical procedure can be successfully applied for various APIs selected for quantitative testing after risk assessment.

## 2. Results and Discussion

### 2.1. Risk Assessment

**Figure 2:**
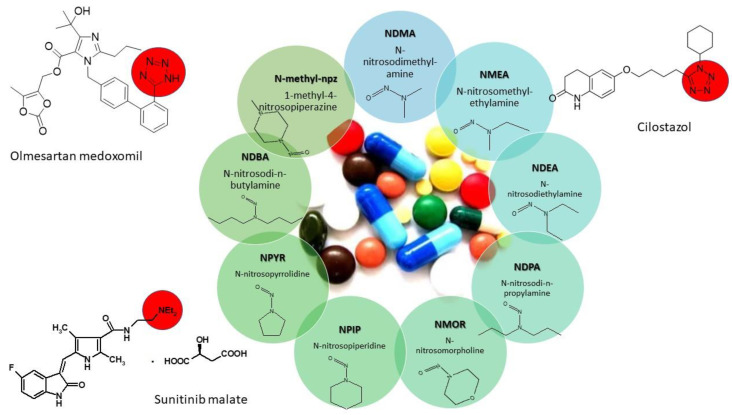
Structures of the APIs and nitrosamines presented in this study. Red circles—types of nitrosamine-related substructures present in APIs.

Figure 2 presents the structures of tested APIs and N-nitrosamines examined in this study.

Since 2019, national regulatory agencies (US FDA, EMA) have obligated every marketing authorization holder to review their manufacturing process in order to identify the risk of nitrosamine formation or cross-contamination and, if necessary, to perform confirmatory testing. Kao et al. [27] in their recent study reported a substructure-based approach as a method for screening and investigating nitrosamines in medical products. According to their work, there are eight substructures present in APIs: DBA (dibutylamine), DEA (dietylamine), DIPA (diisopropylamine), DMA (dimethylamine), IPEA (isopropylethylamine), MPA (methylphenulamine, NMP (N-methyl-2-pyrrolidone), and a tetrazole ring can be suspected to be precursors of nitrosamines. A tetrazole ring is present in the structure of olmesartan medoxomil and cilostazol, while DEA in sunitinib malate (Figure 2). Another risk factor for nitrosamine contamination of sunitinib malate is the use of pyrrolidine in the synthesis. Many suppliers of raw materials for the synthesis of APIs do not provide information about the reagents used in the synthesis, which is why it is so important to selectively check batches with risk elements. Regulatory agencies have implemented analytical methods for sartans, antihistamines, and antidiabetic drugs, but they also continue to test other drug products; so, our new, sensitive screening method for determination of the nine nitrosamines in cilostazol and sunitinib malate is a very useful and suitable tool.

### 2.2. Method Development

A key parameter in the development of the microextraction method for the samples was optimization of the sample solvent. The solubility studies of olmesartan medoxomil, sunitinib malate, and cilostazol are presented in Supplementary Tables S1–S3. The choice of solvent involved the selection of the medium in which centrifugal extraction of the analytes under study was most efficient. Finally, methanol was chosen as the solvent in which cilostazol, olmesartan medoxomil, and sunitinib malate were slightly soluble. During method development, the centrifuge speed, centrifugation time, and amount of solvent taken for extraction were optimized. The best sensitivity was obtained for a centrifugation speed of 15,000 rpm, time of 10 min, and 250 µL of solvent. At lower centrifugation speeds and shorter times (5000 rpm, 10,000 rpm, 2 and 5 min), there was no complete extraction of the analytes (Table S4). At longer centrifugation times, no increase in method sensitivity was observed. During the extraction, no evaporation of the solvent in the centrifuge chamber was noticed. From the capillary columns commonly used in the analysis of nitrosamines, such as DB-5 ms, DB-624 ms, and VF-WAXms [28,29,30], the best chromatographic separation was obtained on the column VF-WAXms (30 m × 0.32 mm, film thickness of 1.0 µm). For the quantification analysis on a single quadruple mass spectrometer, we used electron ionization in selected ion monitoring mode (SIM) of particular ions, ensuring the highest sensitivity was chosen (see Section 3.2).

### 2.3. Validation Results

During the development of the method, satisfactory separation of nine nitrosamines was obtained in selected APIs. The standard added to the sample did not cause peaks splitting and the matrix did not affect the result (Figures S1–S27). Figure 3 shows a chromatogram of the standard solution.

Results for linearity, limit of detection (LOD), and LOQ are summarized in Table 1. The determination coefficients (R2) of the linear regression for the nine nitrosamines were over 0.995, which clearly demonstrates that the method was suitable for quantitative analysis.

The LODs and LOQs calculated both ways from the linearity and ratio peak/noise height were very similar. LODs were determined to be within the range 0.15–1.00 ppb (Table 2); such low LODs are comparable to the results obtained with GC-MS/MS by Liu at al. [11] (Table S5).

The accuracy of the method was verified at three levels—80%, 100%, and 120%—of the specification limit. The results for accuracy for all tested APIs are summarized in Table 2. The results exhibited that recoveries for all nitrosamines in sunitinib malate ranged from 94.09% to 111.22%, for cilostazol from 95.91% to 104.87%, and for olmesartan medoxomil from 92.20% to 108.72%, respectively.

Method precision was evaluated by repeatability, intermediate precision, and system precision. As summarized in Table 3, this GC-MS method exhibited satisfactory RSD% values for repeatability in all APIs in the range of 0.76–5.75%, and for intermediate precision and system precision in the range of 1.13–7.65% and 1.03–3.65% (area), respectively. Since the acceptance criteria for recovery and precision for all APIs were established for 60–115% and RSD < 21%, all the results meet the criteria according to the Association of Official Analytical Chemists [31].

The robustness of the GC-MS method was evaluated on standard solution to determine the impact on the result in case of small variations in the chromatographic parameters: oven initial column temperature ± 5 °C; first temperature ramp ± 1 °C/min; and gas pressure ± 10%. The robustness results (Table S6) showed that the retention time for all nitrosamines change within a range of ± 1.1 min and the separation of the nitrosamines was not affected.

Moreover, during validation, the stability of the standard solution and sample solution was investigated. Standard solution stability studies for all nitrosamines in methanol demonstrated that after 24 and 48 h in 25 °C, the RSD% (area) ranged from 0.63 to 4.79%, demonstrating that the standard solution was stable up to 48 h after preparation (Tables S7–S15). Stability of the sample solution of cilostazol, olmesartan medoxomil, and sunitinib malate spiked with nitrosamines at 100% of a specification level was evaluated after 24 h in 25 °C by measuring the peak area quotient of nitrosamines and NDMA-d6. The RSD% for all the nitrosamines in olmesartan medoxomil was in range of 1.36–18.33%, in cilostazol, 0.69–9.14%, and in sunitinib malate, 0.38–10.63%, which indicated the increase in RSD with time and the necessity of sample solution preparation directly before analysis (Tables S16–S24).

Supplementary Material Table S5 shows the comparison of the results of this publication with various reported GC-MS and GC-MS/MS methods for the detection of N-nitrosamines. The novel GC-MS method with microextraction showed superior results in the validation test compared with other GC-MS and GC-MS/MS methods. The developed method offers several benefits over previously established methodologies, including enhanced sensitivity (LOD below 1 ppb), with no requirements for derivatization and multistep sample preparation (SPE, SPME or DLLEM); a reduction in solvent usage (only 250 µL); and application to a wide variety of APIs. We have also demonstrated that the developed method using a single quadrupole is as good as the method with tandem mass spectrometry.

### 2.4. Application of the Method for Nitrosamine Determination in Selected APIs

The validated GC-MS method was applied to determine nine nitrosamines in three active pharmaceutical ingredients: cilostazol, sunitinib malate, and olmesartan medoxomil. In cilostazol and sunitinib, no nitrosamines were found. NDEA was detected in olmesartan medoxomil at a concentration of 1.66 ng/mL. Therefore, based on the proposed European Pharmacopoeia Commission limit (30 ppb), the acceptance criteria for nitrosamines in APIs have been meet. Olmesartan medoxomil have never been recalled from the market due to detection of nitrosamines beyond acceptable intake limits [2].

## 3. Materials and Methods

### 3.1. Materials

Cilostazol (99.8%), sunitinib malate (99.7%), and olmesartan medoxomil (99.7%) were purchased from Pharmaceutical Research Institute (Poland). The nitrosamines mix (containing NDMA, NMEA, NDEA, NDPA, NMOR, NPYR, NPIP, and NDBA) EPA 8270 (Appendix IX), certified reference material; 2000 µg/mL in methanol) was obtained from Sigma-Aldrich (USA). 1-Methyl-4-nitrosopiperazine was obtained from Angene International Limited (London, England). N-Nitrosodimethylamine-d6 (1000 µg/mL in methanol) was purchased from LGC (Kiełpin, Poland). Methanol LC-MS reagent was obtained from J.T Baker (Oslo, Norway).

### 3.2. Equipment and Method

The GC-MS analysis was performed using a Shimadzu gas chromatograph GCMS-QP2010 ULTRA with MS detector. The separation of nitrosamines was performed on a VF-WAXms polyethylene glycol column (30 m × 0.32 mm, film thickness of 1.0 µm) from Agilent Technologies. In the study, centrifuge tubes with a 0.2 µm PVDF filter from Anchem and an M-Science centrifuge from MPW Med. Instruments were used. All the GC-MS method’s experimental conditions are presented in Table 4. The ions selected for each nitrosamine for quantification and confirmation are shown in Table 5.

### 3.3. Standard Solution Preparation

The internal standard (IS) solution (70 ng/mL in methanol) was prepared from the internal stock solution NDMA-d6 (1000 µg/mL in methanol) and diluted with methanol. The nitrosamine intermediate dilution was prepared by dissolving the appropriate amount of the nitrosamines mix (certified reference material) to reach 10 µg/mL of the nitrosamine mix in MeOH. A standard solution of nine nitrosamines at the concentration level of 18.0 ng/mL was prepared from the nitrosamine intermediate dilution in a 10 mL volumetric flask with methanol.

### 3.4. Sample Solution Preparation

A total of 150 mg of API was weighed into 2 mL centrifuge tubes with filtration equipment and 250 µL of the internal standard solution (70 ng/mL NDMA-d6) was added. Then, the solution was centrifuged at 15,000 rpm for 10 min. A clear organic phase was transferred to a sample vial because the selected APIs were practically insoluble in methanol.

### 3.5. Blank Preparation

The blank solution was prepared as described in Section 3.4., but without addition of the sample.

### 3.6. Validation of the GC-MS Method

The GC-MS method for the nine nitrosamines in API was validated according to ICH guidelines. The specificity of the method was examined using a blank solution, standard solution, sample solution, and reference solution (sample solution spiked with standard solution at a 100% concentration level of the specified limit).

The calibration curve was prepared in methanol at concentrations from LOQ to 120% (0.45–21.6 ng/mL) of the specified limit for nitrosamines with respect to sample preparation, the internal standard solution was added in a concentration of 70 ng/mL NDMA-d6. The linearity for every nitrosamine was evaluated by linear regression and statistical evaluation. Linearity experiments included the determination coefficient, y-intercept, slope of regression line, and residual sum of squares (Sr). The determination coefficients (R2) of the linear regression for each calibration curve should be ≥0.995.

The accuracy of the method was established on three different concentrations: a sample spiked with nitrosamines at 80%, 100%, and 120% of the specification limit; the acceptance criteria were 60–115% and the precision at each concentration should not exceed RSD < 21%. In 2020, the European Pharmacopoeia Commission adopted a new general chapter for trace-level analysis of nitrosamine impurities where the recommended maximum allowed limit for nitrosamines in active substances was set at 0.03 ppm (30 ppb) [32]; therefor, in the present study, the same limit was implemented.

Limit of detection (LOD) and limit of quantitation (LOQ) were calculated in two different ways: using peak height (S) and noise height (N), where the LOQ was stated as S/N ≥ 10 and LOD as S/N ≥ 3, and using linearity parameters such as the standard deviation of the response (σ) and the slope of the curve (S); then, LOD and LOQ were determined from the following equations: LOD = (3.3 × σ/S) and LOQ = (10 × σ/S).

The precision of the method was examined as repeatability, intermediate precision, and system precision. Repeatability for all nitrosamines was obtained from analysis of the six sample solutions. Intermediate precision was assessed as a result of the intra-laboratory variations, i.e., a different day or analyst (the RSD should not exceed 21%), while the difference between the results were tested by statistical Horwitz`s test. System precision was evaluated by six replicate injections of the standard solution (calculation—Supplementary Note S1).

During validation, the stability of the solutions and robustness of the method also were evaluated. The stability of the solutions was established by measuring the peak area quotient of the nitrosamines and NDMA-d6 for the reference solution, with nitrosamines at a 100% concentration level of the specified limit and for a sample solution of API (cilostazol, sunitinib, and olmesartan medoxomil) spiked with nitrosamines at 100% of the specification level (18.0 ng/mL) after 24 h under autosampler conditions (20 °C ± 5 °C). Robustness of the developed method was investigated by injecting the standard solution in different chromatographic conditions: column temperature, ±5 °C; rate, ±1 °C/min; and carrier gas pressure, ±10%.

## 4. Conclusions

A new, sensitive, simple, environmentally friendly method for extracting nine nitrosamines from active pharmaceutical ingredients, using the high-pressure direct injection GC-MS method, was developed in this study. Based on the performed risk evaluation in the manufacturing process, three APIs were examined. This is the first described method for the determination of nitrosamines in cilostazol and sunitinib malate. The implemented GC-MS method for cilostazol and sunitinib malate showed no influence of the manufacturing process on the development of nitrosamine impurities. The quantitative method for each API was validated according to the requirements of the ICH guideline. The validation of the GC-MS method proved that the method was sensitive, accurate, precise, and suitable for nitrosamine determination in multiple APIs. The results obtained during the validation showed that the LOD and LOQ were in the range from 0.15 to 1.00 ng/mL. In conclusion, the presence of nine nitrosamines was determined as below the acceptable intake limits in selected APIs; however, it is necessary to test other APIs containing specific amine substructures in their structures to ensure the safety of the drugs for patients.

## Figures and Tables

**Figure 3:**
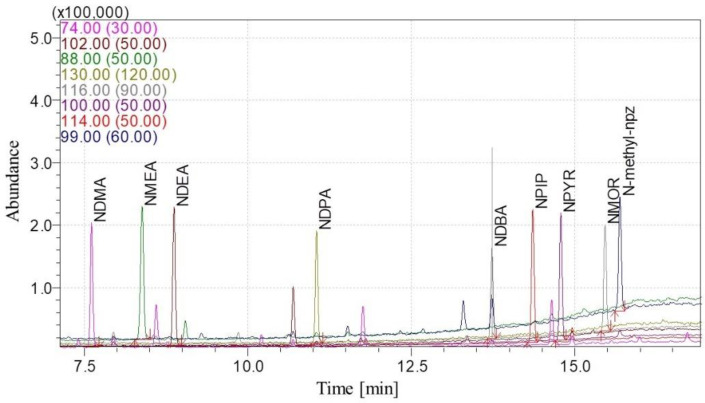
SIM mode chromatogram of the N-nitrosamines standard—18 ng/mL.

**Table 1:** Validation results for linearity, LOD, and LOQ.

Analyte	Range (ng/mL)	*Y = ax + b (R^2^ ≥ 0.990) ** t_a_ ** t_b_	LOD (ng/mL)		LOQ (ng/mL)	
			S/N ≥ 3	(3.3× σ/S)	S/N ≥ 10	(10× σ/S)
NDMA	0.45–21.6	Y = 0.0127x + 0.0005 (R^2^ = 0.9996) 158.53 0.5695	0.15	0.47	0.45	1.43
NMEA	1.5–21.6	Y = 0.0104x + 0.0011 (R^2^ = 0.9997) 159.85 1.4050	0.50	0.40	1.50	1.20
NDEA	1.0–21.6	Y = 0.0088x + 0.0009 (R^2^ = 0.9997) 153.81 1.3608	0.30	0.45	1.00	1.37
NDPA	3.0–21.6	Y = 0.0029x + 0.0007 (R^2^ = 0.9988) 70.521 1.4061	1.00	0.78	3.00	2.38
NMOR	3.0–21.6	Y = 0.0039x−0.0002 (R^2^ = 0.9991) 83.655 0.7650	1.00	0.67	3.00	2.03
NPYR	1.5–21.6	Y = 0.0085x + 0.0007 (R^2^ = 0.9994) 105.24 0.7193	0.50	0.60	1.50	1.82
NPIP	1.5–21.6	Y = 0.0087x + 0.0005 (R^2^ = 0.9992) 91.615 0.4482	0.50	0.69	1.50	2.10
NDBA	1.5–21.6	Y = 0.0032x + 0.0002 (R^2^ = 0.9986) 71.346 0.4260	0.50	0.88	1.5	2.66
*N*-methyl-npz	1.5–21.6	Y = 0.0065x + 0.0013 (R^2^ = 0.9996) 137.02 2.2741	0.50	0.46	1.50	1.40

* a—slope; b—y-intercept. ** t_a_, t_b_—parameters of the Student’s *t*-test; t_b_ = b/S_b_, t_a_ = a/S_a_, where S_a_—the standard deviation of a, and S_b_—the standard deviation of b.

**Table 2:** Validation results of the accuracy for sunitinib, cilostazol, and olmesartan medoxomil.

Analyte	Accuracy Mean Recovery 60–115% RSD ≤ 21% (*n* = 9)		
	Sunitinib Malate	Cilostazol	Olmesartan Medoxomil
NDMA	94.45	98.26	104.66
	1.98	2.11	5.68
NMEA	94.09	99.14	95.85
	2.10	1.78	4.24
NDEA	97.97	97.15	97.34
	3.57	2.02	4.44
NDPA	97.07	95.91	92.20
	5.05	2.15	6.50
NMOR	111.22	97.10	103.60
	2.89	3.66	5.16
NPYR	95.87	100.62	108.72
	3.82	3.00	5.29
NPIP	99.37	96.66	ND
	5.13	6.02	
NDBA	99.30	104.87	102.17
	6.10	2.38	8.98
*N*-methyl-npz	102.94	99.90	ND
	7.53	5.17	

ND—not detected.

**Table 3:** Validation results of precision for sunitinib malate, cilostazol, and olmesartan medoxomil.

	Matrix	Sunitinib		Cilostazol		Olmesartan Medoxomil		System Precision	
Analyte		Repeatability RSD (%)	Intermediate Precision RSD (%)	Repeatability RSD (%)	Intermediate Precision RSD (%)	Repeatability RSD (%)	Intermediate Precision RSD (%)	Area Nitrosamine/Area NDMA-d6 RSD (%)	Retention Time RSD (%)
NDMA		2.12	2.05	0.76	1.13	3.07	2.51	1.31	0.03
NMEA		2.25	1.99	1.27	1.42	2.47	2.21	1.08	0.02
NDEA		2.48	3.37	1.10	1.37	3.00	4.04	1.31	0.02
NDPA		3.95	6.26	2.97	2.25	5.75	4.24	1.03	0.01
NMOR		3.18	5.55	2.73	4.49	3.07	3.97	3.52	0.01
NPYR		2.51	5.08	1.77	3.91	2.55	7.50	2.42	0.01
NPIP		3.67	4.06	3.91	4.60	ND	ND	2.01	0.01
NDBA		4.32	6.81	1.68	1.46	4.51	4.71	3.07	0.01
*N*-methyl-npz		2.22	6.45	5.60	7.65	ND	ND	3.65	0.01

ND—not detected.

**Table 4:** GC-MS method experimental conditions.

GC Parameters	
Carrier gas	Helium
Column flow rate	2.1 mL/min
Oven initial temperature	75 °C (hold time: 2 min)
First temperature ramp	9.0 °C/min
Second temperature	200 °C/min (hold time: 2 min)
Second temperature ramp	15.0 °C/min
Final temperature	240 °C (hold time: 3 min)
Pressure	45.5 kPa
Injection mode	Splitless, high-pressure injection 130 kPa
Injection port temperature	150 °C
**MS Parameters**	
Ion Source Temperature	240
Interface Temperature	240
Acquisition type	SIM
Solvent delay	6.5 min

**Table 5:** GC-MS SIM for the detected nitrosamines.

Compound	Quantification Ion (*m*/*z*)	Confirmation Ion (*m*/*z*)
NDMA	74	44
NMEA	88	56
NDEA	102	56
NDPA	130	70
NMOR	116	56
NPYR	100	68
NPIP	114	55
NDBA	116	158, 84
*N*-methyl-npz	99	100, 56
NDMA-d6	80	50

## Data Availability

Not applicable.

## Supplementary Materials

The following supporting information can be downloaded at: https://www.mdpi.com/article/10.3390/ijms232012125/s1.
