# Supplementary material for: Development of a Sensitive Screening Method for Simultaneous Determination of Nine Genotoxic Nitrosamines in Active Pharmaceutical Ingredients by GC-MS
Source: Int J Mol Sci. 2022 Oct 12;23(20):12125. doi: 10.3390/ijms232012125 (PMC9603764; doi:10.3390/ijms232012125)
Supplement: Supplementary file 1 [file ijms-23-12125-s001.zip › ijms-1920422-supplementary.pdf]

Supplementary Material

Development of a sensitive screening method for simultaneous determination of nine genotoxic nitrosamines in active pharmaceutical ingredients by GC-MS.

Anna B. Witkowska [1,2*], Joanna Giebułtowicz[3], Magdalena Dąbrowska[1] and Elżbieta U. Stolarczyk [1,4]

List of Figures, Tables and Notes:

TABLE S1. RESULTS OF SOLUBILITY ANALYSES OF SUNITINIB MALATE. 4
TABLE S2.RESULTS OF SOLUBILITY ANALYSES OF OLMESARTAN MEDOXOMIL. 5
TABLE S3.RESULTS OF SOLUBILITY ANALYSES OF CILOSTAZOL. 5
TABLE S4.EXPERIMENTAL DESIGN FOR OPTIMIZING THE EXTRACTION CONDITIONS. 6
TABLE S5.COMPARISON OF THE RESULTS OF THIS PUBLICATION WITH VARIOUS REPORTED GC-MS AND GC-MS/MS METHODS FOR THE DETECTION OF NITROSAMINES. 6
TABLE S6.THE RESULTS OF THE ROBUSTNESS FOR NINE NITROSAMINES. 9
TABLE S7.RESULTS OF SYSTEM PRECISION FOR NDMA. 10
TABLE S8.RESULTS OF SYSTEM PRECISION FOR NMEA. 10
TABLE S9.RESULTS OF SYSTEM PRECISION FOR NDEA. 11
TABLE S10.RESULTS OF SYSTEM PRECISION FOR NDPA. 11
TABLE S11.RESULTS OF SYSTEM PRECISION FOR NMOR. 12
TABLE S12.RESULTS OF SYSTEM PRECISION FOR NPYR. 13
TABLE S13.RESULTS OF SYSTEM PRECISION FOR NPIP. 13
TABLE S14.RESULTS OF SYSTEM PRECISION FOR NDBA. 14
TABLE S15.RESULTS OF SYSTEM PRECISION FOR N-METHYL-NPZ. 14
TABLE S16.RESULTS OF EXAMINED SAMPLE SOLUTION WITH NDMA AT 100% CONCENTRATION LEVEL. 15
TABLE S17.RESULTS OF EXAMINED SAMPLE SOLUTION WITH NMEA AT 100% CONCENTRATION LEVEL. 15
TABLE S18.RESULTS OF EXAMINED SAMPLE SOLUTION WITH NDEA AT 100% CONCENTRATION LEVEL. 16
TABLE S19.RESULTS OF EXAMINED SAMPLE SOLUTION WITH NDPA AT 100% CONCENTRATION LEVEL. 16
TABLE S20.RESULTS OF EXAMINED SAMPLE SOLUTION WITH NMOR AT 100% CONCENTRATION LEVEL. 17
TABLE S21.RESULTS OF EXAMINED SAMPLE SOLUTION WITH NPYR AT 100% CONCENTRATION LEVEL. 17
TABLE S22.RESULTS OF EXAMINED SAMPLE SOLUTION WITH NPIP AT 100% CONCENTRATION LEVEL. 18

TABLE S23.RESULTS OF EXAMINED SAMPLE SOLUTION WITH NDBA AT 100% CONCENTRATION LEVEL. 19
TABLE S24.RESULTS OF EXAMINED SAMPLE SOLUTION WITH N-METHYL-NPZ AT 100% CONCENTRATION LEVEL. 19
FIGURE S1. COMPARISON OF CHROMATOGRAMS FOR NDMA M/Z 74 A) BLANK (BLACK LINE) B) SAMPLE SOLUTION OF SUNITINIB (PINK LINE) C) REFERENCE SAMPLE SOLUTION - SUNITINIB SPIKED WITH THE STANDARD SOLUTION OF NITROSAMINES AT CONCENTRATION LEVEL OF 18.0 NG/ML (BLUE LINE) (CAL 100%). 20
FIGURE S2. COMPARISON OF CHROMATOGRAMS FOR NMEA M/Z 88 A) BLANK (BLACK LINE) B) SAMPLE SOLUTION OF SUNITINIB (PINK LINE) C) REFERENCE SAMPLE SOLUTION SUNITINIB SPIKED WITH THE STANDARD SOLUTION OF NITROSAMINES AT CONCENTRATION LEVEL OF 18.0 NG/ML (BLUE LINE) (CAL 100%). 20
FIGURE S3. COMPARISON OF CHROMATOGRAMS FOR NDEA M/Z 102 A) BLANK (BLACK LINE) B) SAMPLE SOLUTION OF SUNITINIB (PINK LINE) C) REFERENCE SAMPLE SOLUTION SUNITINIB SPIKED WITH THE STANDARD SOLUTION OF NITROSAMINES AT CONCENTRATION LEVEL OF 18.0 NG/ML (BLUE LINE) (CAL 100%). 21
FIGURE S4. COMPARISON OF CHROMATOGRAMS FOR NDPA M/Z 130 A) BLANK (BLACK LINE) B) SAMPLE SOLUTION OF SUNITINIB (PINK LINE) C) REFERENCE SAMPLE SOLUTION SUNITINIB SPIKED WITH THE STANDARD SOLUTION OF NITROSAMINES AT CONCENTRATION LEVEL OF 18.0 NG/ML (BLUE LINE) (CAL 100%). 21
FIGURE S5. COMPARISON OF CHROMATOGRAMS FOR NMOR M/Z 116 A) BLANK (BLACK LINE) B) SAMPLE SOLUTION OF SUNITINIB (PINK LINE) C) REFERENCE SAMPLE SOLUTION SUNITINIB SPIKED WITH THE STANDARD SOLUTION OF NITROSAMINES AT CONCENTRATION LEVEL OF 18.0 NG/ML (BLUE LINE) (CAL 100%). 22
FIGURE S6. COMPARISON OF CHROMATOGRAMS FOR NPYR M/Z 100 A) BLANK (BLACK LINE) B) SAMPLE SOLUTION OF SUNITINIB (PINK LINE) C) REFERENCE SAMPLE SOLUTION SUNITINIB SPIKED WITH THE STANDARD SOLUTION OF NITROSAMINES AT CONCENTRATION LEVEL OF 18.0 NG/ML (BLUE LINE) (CAL 100%). 22
FIGURE S7. COMPARISON OF CHROMATOGRAMS FOR NPIP M/Z 114 A) BLANK (BLACK LINE) B) SAMPLE SOLUTION OF SUNITINIB (PINK LINE) C) REFERENCE SAMPLE SOLUTION SUNITINIB SPIKED WITH THE STANDARD SOLUTION OF NITROSAMINES AT CONCENTRATION LEVEL OF 18.0 NG/ML (BLUE LINE) (CAL 100%). 23
FIGURE S8. COMPARISON OF CHROMATOGRAMS FOR NDBA M/Z 158 A) BLANK (BLACK LINE) B) SAMPLE SOLUTION OF SUNITINIB (PINK LINE) C) REFERENCE SAMPLE SOLUTION SUNITINIB SPIKED WITH THE STANDARD SOLUTION OF NITROSAMINES AT CONCENTRATION LEVEL OF 18.0 NG/ML (BLUE LINE) (CAL 100%). 23
FIGURE S9. COMPARISON OF CHROMATOGRAMS FOR N-METHYL-NPZ M/Z =99 A) BLANK (BLACK LINE) B) SAMPLE SOLUTION OF SUNITINIB (PINK LINE) C) REFERENCE SAMPLE SOLUTION SUNITINIB SPIKED WITH THE STANDARD SOLUTION OF NITROSAMINES AT CONCENTRATION LEVEL OF 18.0 NG/ML (BLUE LINE) (CAL 100%). 24
FIGURE S10. COMPARISON OF CHROMATOGRAMS FOR NDMA M/Z 74 A) BLANK (BLACK LINE) B) SAMPLE SOLUTION OF CILOSTAZOL (PINK LINE) C) REFERENCE SAMPLE SOLUTION CILOSTAZOL SPIKED WITH THE STANDARD SOLUTION OF NITROSAMINES AT CONCENTRATION LEVEL OF 18.0 NG/ML (BLUE LINE) (CAL 100%). 24

FIGURE S11. COMPARISON OF CHROMATOGRAMS FOR NMEA M/Z 88 A) BLANK (BLACK LINE) B) SAMPLE SOLUTION OF CILOSTAZOL (PINK LINE) C) REFERENCE SAMPLE SOLUTION CILOSTAZOL SPIKED WITH THE STANDARD SOLUTION OF NITROSAMINES AT CONCENTRATION LEVEL OF 18.0 NG/ML (BLUE LINE) (CAL 100%). 25
FIGURE S12. COMPARISON OF CHROMATOGRAMS FOR NDEA M/Z 102 A) BLANK (BLACK LINE) B) SAMPLE SOLUTION OF CILOSTAZOL (PINK LINE) C) REFERENCE SAMPLE SOLUTION CILOSTAZOL SPIKED WITH THE STANDARD SOLUTION OF NITROSAMINES AT CONCENTRATION LEVEL OF 18.0 NG/ML (BLUE LINE) (CAL 100%). 25
FIGURE S13. COMPARISON OF CHROMATOGRAMS FOR NDPA M/Z 130 A) BLANK (BLACK LINE) B) SAMPLE SOLUTION OF CILOSTAZOL (PINK LINE) C) REFERENCE SAMPLE SOLUTION CILOSTAZOL SPIKED WITH THE STANDARD SOLUTION OF NITROSAMINES AT CONCENTRATION LEVEL OF 18.0 NG/ML (BLUE LINE) (CAL 100%). 26
FIGURE S14. COMPARISON OF CHROMATOGRAMS FOR NMOR M/Z 116 A) BLANK (BLACK LINE) B) SAMPLE SOLUTION OF CILOSTAZOL (PINK LINE) C) REFERENCE SAMPLE SOLUTION CILOSTAZOL SPIKED WITH THE STANDARD SOLUTION OF NITROSAMINES AT CONCENTRATION LEVEL OF 18.0 NG/ML (BLUE LINE) (CAL 100%). 26
FIGURE S15. COMPARISON OF CHROMATOGRAMS FOR NPYR M/Z 100 A) BLANK (BLACK LINE) B) SAMPLE SOLUTION OF CILOSTAZOL (PINK LINE) C) REFERENCE SAMPLE SOLUTION CILOSTAZOL SPIKED WITH THE STANDARD SOLUTION OF NITROSAMINES AT CONCENTRATION LEVEL OF 18.0 NG/ML (BLUE LINE) (CAL 100%). 27
FIGURE S16. COMPARISON OF CHROMATOGRAMS FOR NPIP M/Z 114 A) BLANK (BLACK LINE) B) SAMPLE SOLUTION OF CILOSTAZOL (PINK LINE) C) REFERENCE SAMPLE SOLUTION CILOSTAZOL SPIKED WITH THE STANDARD SOLUTION OF NITROSAMINES AT CONCENTRATION LEVEL OF 18.0 NG/ML (BLUE LINE) (CAL 100%). 27
FIGURE S17. COMPARISON OF CHROMATOGRAMS FOR NDBA M/Z 158 A) BLANK (BLACK LINE) B) SAMPLE SOLUTION OF CILOSTAZOL (PINK LINE) C) REFERENCE SAMPLE SOLUTION CILOSTAZOL SPIKED WITH THE STANDARD SOLUTION OF NITROSAMINES AT CONCENTRATION LEVEL OF 18.0 NG/ML (BLUE LINE) (CAL 100%). 28
FIGURE S18. COMPARISON OF CHROMATOGRAMS FOR N-METHYL-NPZ M/Z 99 A) BLANK (BLACK LINE) B) SAMPLE SOLUTION OF CILOSTAZOL (PINK LINE) C) REFERENCE SAMPLE SOLUTION CILOSTAZOL SPIKED WITH THE STANDARD SOLUTION OF NITROSAMINES AT CONCENTRATION LEVEL OF 18.0 NG/ML (BLUE LINE) (CAL 100%). 28
FIGURE S19. COMPARISON OF CHROMATOGRAMS FOR NDMA M/Z 74 A) BLANK (BLACK LINE) B) SAMPLE SOLUTION OF OLMESARTAN MEDOXOMIL (PINK LINE) C) REFERENCE SAMPLE SOLUTION OLMESARTAN MEDOXOMIL SPIKED WITH THE STANDARD SOLUTION OF NITROSAMINES AT CONCENTRATION LEVEL OF 18.0 NG/ML (BLUE LINE) (CAL 100%)). 29
FIGURE S20. COMPARISON OF CHROMATOGRAMS FOR NMEA M/Z 88 A) BLANK (BLACK LINE) B) SAMPLE SOLUTION OF OLMESARTAN MEDOXOMIL (PINK LINE) C) REFERENCE SAMPLE SOLUTION OLMESARTAN MEDOXOMIL SPIKED WITH THE STANDARD SOLUTION OF NITROSAMINES AT CONCENTRATION LEVEL OF 18.0 NG/ML (BLUE LINE) (CAL 100%)). 29
FIGURE S21. COMPARISON OF CHROMATOGRAMS FOR NDEA M/Z 102 A) BLANK (BLACK LINE) B) SAMPLE SOLUTION OF OLMESARTAN MEDOXOMIL (PINK LINE) C) REFERENCE SAMPLE SOLUTION OLMESARTAN MEDOXOMIL SPIKED WITH THE

STANDARD SOLUTION OF NITROSAMINES AT CONCENTRATION LEVEL OF 18. 0 NG/ML (BLUE LINE) (CAL 100%)). 30
FIGURE S22. COMPARISON OF CHROMATOGRAMS FOR NDPA M/Z 130 A) BLANK (BLACK LINE) B) SAMPLE SOLUTION OF OLMESARTAN MEDOXOMIL (PINK LINE) C) REFERENCE SAMPLE SOLUTION OLMESARTAN MEDOXOMIL SPIKED WITH THE STANDARD SOLUTION OF NITROSAMINES AT CONCENTRATION LEVEL OF 18. 0 NG/ML (BLUE LINE) (CAL 100%)). 30
FIGURE S23. COMPARISON OF CHROMATOGRAMS FOR NMOR M/Z 116 A) BLANK (BLACK LINE) B) SAMPLE SOLUTION OF OLMESARTAN MEDOXOMIL (PINK LINE) C) REFERENCE SAMPLE SOLUTION OLMESARTAN MEDOXOMIL SPIKED WITH THE STANDARD SOLUTION OF NITROSAMINES AT CONCENTRATION LEVEL OF 18. 0 NG/ML (BLUE LINE) (CAL 100%)). 31
FIGURE S24. COMPARISON OF CHROMATOGRAMS FOR NPYR M/Z 100 A) BLANK (BLACK LINE) B) SAMPLE SOLUTION OF OLMESARTAN MEDOXOMIL (PINK LINE) C) REFERENCE SAMPLE SOLUTION OLMESARTAN MEDOXOMIL SPIKED WITH THE STANDARD SOLUTION OF NITROSAMINES AT CONCENTRATION LEVEL OF 18. 0 NG/ML (BLUE LINE) (CAL 100%)). 31
FIGURE S25. COMPARISON OF CHROMATOGRAMS FOR NPIP M/Z 114 A) BLANK (BLACK LINE) B) SAMPLE SOLUTION OF OLMESARTAN MEDOXOMIL (PINK LINE) C) REFERENCE SAMPLE SOLUTION OLMESARTAN MEDOXOMIL SPIKED WITH THE STANDARD SOLUTION OF NITROSAMINES AT CONCENTRATION LEVEL OF 18. 0 NG/ML (BLUE LINE) (CAL 100%)). 32
FIGURE S26. COMPARISON OF CHROMATOGRAMS FOR NDBA M/Z 158 A) BLANK (BLACK LINE) B) SAMPLE SOLUTION OF OLMESARTAN MEDOXOMIL (PINK LINE) C) REFERENCE SAMPLE SOLUTION OLMESARTAN MEDOXOMIL SPIKED WITH THE STANDARD SOLUTION OF NITROSAMINES AT CONCENTRATION LEVEL OF 18. 0 NG/ML (BLUE LINE) (CAL 100%)). 32
FIGURE S27. COMPARISON OF CHROMATOGRAMS FOR N-METHYL-NPZ M/Z99 A) BLANK (BLACK LINE) B) SAMPLE SOLUTION OF OLMESARTAN MEDOXOMIL (PINK LINE) C) REFERENCE SAMPLE SOLUTION OLMESARTAN MEDOXOMIL SPIKED WITH THE STANDARD SOLUTION OF NITROSAMINES AT CONCENTRATION LEVEL OF 18. 0 NG/ML (BLUE LINE) (CAL 100%)). 33
SUPPLEMENTARY NOTE S1 – CALCULATIONS 1 33

Table S1. Results of solubility analyses of sunitinib malate.

Solvent	Sample weight [mg]	Volume of solvent added [ml]	Result
water	100 mg	10 ml	Slightly Soluble
methanol	100 mg	10 ml	Slightly Soluble

ethanol	100 mg	10 ml	Slightly Soluble
Propan-2-ol	1 mg	>10 ml	Practically insoluble
Hexane	1 mg	>10 ml	Practically insoluble
buffer pH 1,2	100 mg	3 ml	Soluble
buffer pH 7,2	100 mg	3 ml	Soluble
buffer pH 9,2	100 mg	3 ml	Soluble
buffer pH 9,5	100mg	10ml	Slightly Soluble

Table S2.Results of solubility analyses of olmesartan medoxomil.

Solvent	Sample weight [mg]	Volume of solvent added [ml]	Result
**water**	<1	10	Practically insoluble
**etanol**	10	10	slightly soluble
**heptane**	<1	10	Practically insoluble
**acetonitrile**	10	10	slightly soluble
**methanol**	10	10	slightly soluble
**DMSO**	100	1	freely soluble

Table S3.Results of solubility analyses of cilostazol.

Solvent	Sample weight [mg]	Volume of solvent added [ml]	Result
water	<1	10	Practically insoluble
methanol	10	10	slightly soluble
2-propanol	1	10	Very Slightly Soluble
Acetic acid	100	1	Freely soluble
DMSO	100	3	soluble
hexan	<1	10	Practically insoluble

acetonitrile	10	10	slightly soluble
buffet pH 1,23	<1	10	Practically insoluble
buffer pH 4,58	<1	10	Practically insoluble
buffer pH 7,25	<1	10	Practically insoluble
buffer pH 9,5	<1	10	Practically insoluble

Table S4.Experimental design for optimizing the extraction conditions.

Experiment No.	methanol	Centrifuge Speer [rpm]	Centrifuge time [min]	Result Mean Recovery* [%]
1	250µL	15000	2	volume too small for GC injection
2	250µL	15000	5	98.76% about 100ul extracted
3	250uL	15000	7	99.07% about 150ul extracted
4	250ul	15000	10	99.38% about 220 ul extracted
4	250µL	5000	10	volume too small for GC injection
5	250µL	10000	10	volume too small for GC injection

*Recovery = (Area nitrosamine /Area NDMA-d6 for sample solution with nitrosamine at 100% concentration level after extraction)/(Area nitrosamine /Area NDMA-d6 for nitrosamine at 100% of the specification limit)

Table S5.Comparison of the results of this publication with various reported GC-MS and GC-MS/MS methods for the detection of nitrosamines.

No.	Method / API	Nitrosamines	Linearity/ Range	Accuracy	Precision	LOD/LOQ [ppb]	Ref.
1	HS-SPME-GC-MS/ ranitidine	NDMA	R: 0.998 5–100 ppb	-	RSD=20% (5ppb)	LOD=1 LOQ=5	[1][1]
2	DLLME-GC-MS/ ranitidine	NDMA NEMA NDEA NPYR NMOR NDPA NPIP NDBA NDPhA	R^2>0.99 NDMA :30-3000 ppb NMOR:15-3000 ppb NEMA. NPYR:3-3000 ppb NDEA. NDPA. NPIP. NDBA:0.5-3000 ppb	80.2 and 102% (n=36) for 50 ppb	RSD<12% (reproducibility) RSD<10% (repeatability)	LOQ_{NDMA}=21 LOQ_{NMEA}=11 LOQ_{NDEA}=0.96 LOQ_{NPYR}=8.4 LOQ_{NMOR}=17 LOQ_{NDPA}=1.1 LOQ_{NPIP}=1.6 LOQ_{NDBA}=1.1 LOQ_{NDPhA}=0.21	[2]
3	GC-MS/MS/ sartans	NDMA NDEA NDBA NDIPA	R^2>0.99 NDMA: 0.8-60 ppb NDEA: 0.8-16 ppb NDIPA: 3-60 ppb NDBA: 6-60 ppb	87.68-123.76%	Intraday: 1.45-6.38% Interday: 2.88-9.15%	LOD=2 -150 LOQ= 8-500	[3]

4	SPE-GC-MS/MS/ sartans, ranitidine and metformin	NDMA NDEA	R^2=1.000 NDEA: 0.3-1000 ppb NDMA: 0.9-1000 ppb	NDMA: 95-105% NDEA: 93.6-104%	RSD_{NDEA}: 0.4-4.2% RSD_{NDMA}: 0.4-2.7%	LOQ= 0.3-0.9 ppb	[4]
5	GC-MS/Valsartan	NDMA	R>0.999 5–200ppb	-	RSD: 1.9% (n=5)	LOD=1ppb	[5]
6	HS-GC-MS/MS /valsartan	NDMA NDEA NEIPA NDIPA	R≥0.995	-	-	LOD_{NDMA}=10 LOD_{NDEA}=10 LOD_{NEIPA}=25 LOD_{NDIPA}=25	[6]
7	GC-MS/ranitidine	NDMA	R^2 = 0.99986	-	-	LOD_{NDMA}=5.9 LOQ_{NDMA}=19.8	[7]
8	HS-GC-MS/losartan	NDMA NDEA EIPNA DIPNA	R^2>0.999 25-5000ppb	-	RSD<11.5%	$LOQ_{NDMA.NDEA}$=25 $LOQ_{EIPNA.DIPNA}$=50	[8]
9	SF-HS-GC/MS/ Drug products	NDMA	R>0.9999 5-500ppb	92.77-106.54%	RSD:1.20-5.94%	LOQ=5	[9]

	/ranitidine						
10	GC-MS /cilostazol,sunitinib and olmesartan	NDMA<br>NMEA<br>NDEA<br>NDPA<br>NMOR<br>NPYR<br>NPIP<br>NDBA	R^2>0.995<br>0.15-21.6 ppb	94.09-111.22%	RSD<7.65%	LOD:0.15-1ppb	This work

Table S6.The results of the robustness for nine nitrosamines.

Changed parameters	Retention time (min)								
	NDMA	NMEA	NDEA	NDPA	NMOR	NPYR	NPIP	NDBA	N-methyl-npz
Main method	7.611	8.383	8.872	11.051	15.464	14.786	14.358	13.733	15.690
Column temperature 75→70°C	8.140	8.914	9.405	11.597	16.017	15.339	14.909	14.287	16.244
Column temperature 75→80°C	7.082	7.852	8.339	10.506	14.909	14.234	13.807	13.183	15.139
Rate 8°C/min	7.894	8.737	9.273	11.683	16.579	15.814	15.329	14.668	16.822
Rate 10°C/min	7.310	8.074	8.525	11.519	14.536	13.930	13.547	12.955	14.758
Carrier gas pressure 41 kPa	7.724	8.501	8.992	11.177	15.600	14.926	14.498	13.866	15.831

Carrier gas pressure 50 kPa	7.498	8.268	8.754	10.928	15.331	14.653	14.223	13.611	15.556

Table S7.Results of system precision for NDMA.

	Solution with NDMA at 100% of the specification limit	
	Area NDMA/Area NDMA-d6 (Q)	Retention time (min)
1	0.224950	7.610
2	0.224810	7.611
3	0.224619	7.608
4	0.229979	7.606
5	0.231007	7.610
6	0.229457	7.607
Mean	0.227	7.609
SD	0.003	0.002
RSD%	1.309	0.026

Table S8.Results of system precision for NMEA.

	Solution with NMEA at 100% of the specification limit	
	Area NMEA/Area NDMA-d6 (Q)	Retention time (min)

1	0.186375	8.383
2	0.187098	8.383
3	0.192178	8.381
4	0.188010	8.379
5	0.188924	8.382
6	0.189251	8.380
Mean	0.189	8.381
SD	0.002	0.002
RSD%	1.083	0.019

Table S9.Results of system precision for NDEA.

	Solution with NDEA at 100% of the specification limit	
	Area NDEA/Area NDMA-d6 (Q)	Retention time (min)
1	0.157667	8.871
2	0.157493	8.872
3	0.161073	8.870
4	0.161134	8.869
5	0.160501	8.872
6	0.162765	8.869
Mean	0.160	8.871
SD	0.002	0.001
RSD%	1.310	0.016

Table S10.Results of system precision for NDPA.

	Solution with NDPA at 100%

	of the specification limit	
	Area NDPA/Area NDMA-d6	Retention time (min)
1	0.052283	11.051
2	0.051608	11.053
3	0.052613	11.051
4	0.052650	11.049
5	0.052059	11.051
6	0.053165	11.050
Mean	0.052	11.051
SD	0.001	0.001
RSD%	1.028	0.012

Table S11.Results of system precision for NMOR.

	Solution with NMOR at 100% of the specification limit	
	Area NMOR/Area NDMA-d6	Retention time (min)
1	0.067756	15.463
2	0.068651	15.465
3	0.073053	15.464
4	0.069254	15.463
5	0.068313	15.465
6	0.073295	15.462
Mean	0.070	15.464
SD	0.002	0.001
RSD%	3.521	0.008

Table S12.Results of system precision for NPYR.

	Solution with NPYR at 100% of the specification limit	
	Area NPYR/Area NDMA-d6	Retention time (min)
1	0.148465	14.787
2	0.154245	14.787
3	0.158070	14.786
4	0.150725	14.787
5	0.150951	14.787
6	0.156434	14.786
Mean	0.153	14.787
SD	0.004	0.001
RSD%	2.423	0.003

Table S13.Results of system precision for NPIP.

	Solution with NPIP at 100% of the specification limit	
	Area NPIP/Area NDMA-d6	Retention time (min)
1	0.155312	14.358
2	0.158814	14.360
3	0.162168	14.357
4	0.159895	14.359
5	0.158157	14.359
6	0.164522	14.356

Mean	0.160	14.358
SD	0.003	0.001
RSD%	2.013	0.010

Table S14.Results of system precision for NDBA.

	Solution with NDBA at 100% of the specification limit	
	Area NDBA/Area NDMA-d6	Retention time (min)
1	0.054702	13.733
2	0.057474	13.736
3	0.058570	13.735
4	0.057383	13.734
5	0.055618	13.735
6	0.059367	13.733
Mean	0.057	13.734
SD	0.002	0.001
RSD%	3.071	0.009

Table S15.Results of system precision for N-methyl-npz.

	Solution with NDMA at 100% of the specification limit	
	Area NDMA/Area	Retention time (min)

	NDMA-d6	
1	0.115483	15.691
2	0.117125	15.693
3	0.125361	15.689
4	0.114147	15.691
5	0.122528	15.692
6	0.120568	15.689
Mean	0.119	15.691
SD	0.004	0.002
RSD%	3.648	0.010

Table S16.Results of examined sample solution with NDMA at 100% concentration level.

API	Olmesartan medoxomil	Sunitinib malate	Cilostazol
Time	Area NDMA/Area NDMA-d6	Area NDMA/Area NDMA-d6	Area NDMA/Area NDMA-d6
0 h	0.33764	0.21966	0.22978
0 h	0.33193	0.22031	0.22868
24 h	0.36238	0.22120	0.23358
24 h	0.36151	0.21931	0.22935
Mean	0.34836	0.22012	0.23035
SD	0.01586	0.00083	0.00220
RSD%	4.55208	0.37721	0.95497

Table S17.Results of examined sample solution with NMEA at 100% concentration level.

API	Olmesartan medoxomil	Sunitinib malate	Cilostazol

Time	Area NMEA/Area NDMA-d6	Area NMEA/Area NDMA-d6	Area NMEA/Area NDMA-d6
0 h	0.25164	0.18081	0.18665
0 h	0.24375	0.17944	0.18496
24 h	0.24736	0.18567	0.18353
24 h	0.24555	0.18362	0.18504
Mean	0.24708	0.18238	0.18504
SD	0.00338	0.00280	0.00128
RSD%	1.36784	1.53378	0.69044

Table S18.Results of examined sample solution with NDEA at 100% concentration level.

API	Olmesartan medoxomil	Sunitinib malate	Cilostazol
Time	Area NDEA/Area NDMA-d6	Area NDEA/Area NDMA-d6	Area NDEA/Area NDMA-d6
0 h	0.21025	0.15179	0.15861
0 h	0.21178	0.15428	0.16259
24 h	0.19945	0.16162	0.16569
24 h	0.19923	0.15919	0.17113
Mean	0.20518	0.15672	0.16450
SD	0.00677	0.00449	0.00528
RSD%	3.30020	2.86280	3.21246

Table S19.Results of examined sample solution with NDPA at 100% concentration level.

API	Olmesartan medoxomil	Sunitinib malate	Cilostazol

Time	Area NDPA/Area NDMA-d6	Area NDPA/Area NDMA-d6	Area NDPA/Area NDMA-d6
0 h	0.06580	0.04943	0.05064
0 h	0.06535	0.05042	0.05172
24 h	0.05565	0.05394	0.05714
24 h	0.05746	0.05333	0.05694
Mean	0.06106	0.05178	0.05411
SD	0.00526	0.00219	0.00342
RSD%	8.61696	4.23476	6.31146

Table S20.Results of examined sample solution with NMOR at 100% concentration level.

API	Olmesartan medoxomil	Sunitinib malate	Cilostazol
Time	Area NMOR /Area NDMA-d6	Area NMOR /Area NDMA-d6	Area NMOR /Area NDMA-d6
0 h	0.08685	0.08238	0.07029
0 h	0.08766	0.08049	0.06860
24 h	0.06064	0.09393	0.07726
24 h	0.06674	0.08616	0.07603
Mean	0.07547	0.08574	0.07304
SD	0.01383	0.00595	0.00425
RSD%	18.32974	6.93809	5.81212

Table S21.Results of examined sample solution with NPYR at 100% concentration level.

API	Olmesartan medoxomil	Sunitinib malate	Cilostazol
Time	Area NPYR /Area NDMA-d6	Area NPYR /Area NDMA-d6	Area NPYR /Area NDMA-d6
0 h	0.21536	0.14167	0.16174
0 h	0.18898	0.14495	0.16413
24 h	0.22201	0.17209	0.18963
24 h	0.20827	0.17246	0.18312
Mean	0.20866	0.15779	0.17465
SD	0.01426	0.01678	0.01383
RSD%	6.83651	10.63381	7.91622

Table S22.Results of examined sample solution with NPIP at 100% concentration level.

API	Olmesartan medoxomil	Sunitinib malate	Cilostazol
Time	Area NPIP /Area NDMA-d6	Area NPIP /Area NDMA-d6	Area NPIP /Area NDMA-d6
0 h	ND	0.16373	0.16830
0 h	ND	0.15047	0.15177
24 h	ND	0.16657	0.17921
24 h	ND	0.15242	0.18189
Mean	-	0.15830	0.17029
SD	-	0.00803	0.01368
RSD%	-	5.07573	8.03132

- ND-not detected

Table S23.Results of examined sample solution with NDBA at 100% concentration level.

API	Olmesartan medoxomil	Sunitinib malate	Cilostazol
Time	Area NDBA /Area NDMA-d6	Area NDBA /Area NDMA-d6	Area NDBA /Area NDMA-d6
0 h	0.02726	0.01963	0.02324
0 h	0.02624	0.02067	0.02265
24 h	0.01935	0.02328	0.02659
24 h	0.02090	0.02205	0.02489
Mean	0.02344	0.02141	0.02434
SD	0.00390	0.00160	0.00177
RSD%	16.64789	7.45161	7.27073

Table S24.Results of examined sample solution with N-methyl-npz at 100% concentration level.

API	Olmesartan medoxomil	Sunitinib malate	Cilostazol
Time	Area N-methyl-npz /Area NDMA-d6	Area N-methyl-npz /Area NDMA-d6	Area N-methyl-npz /Area NDMA-d6
0 h	ND	0.12766	0.12340
0 h	ND	0.12205	0.12535
24 h	ND	0.12746	0.14348
24 h	ND	0.13270	0.14754
Mean	-	0.12747	0.13494
SD	-	0.00435	0.01234
RSD%	-	3.41481	9.14427

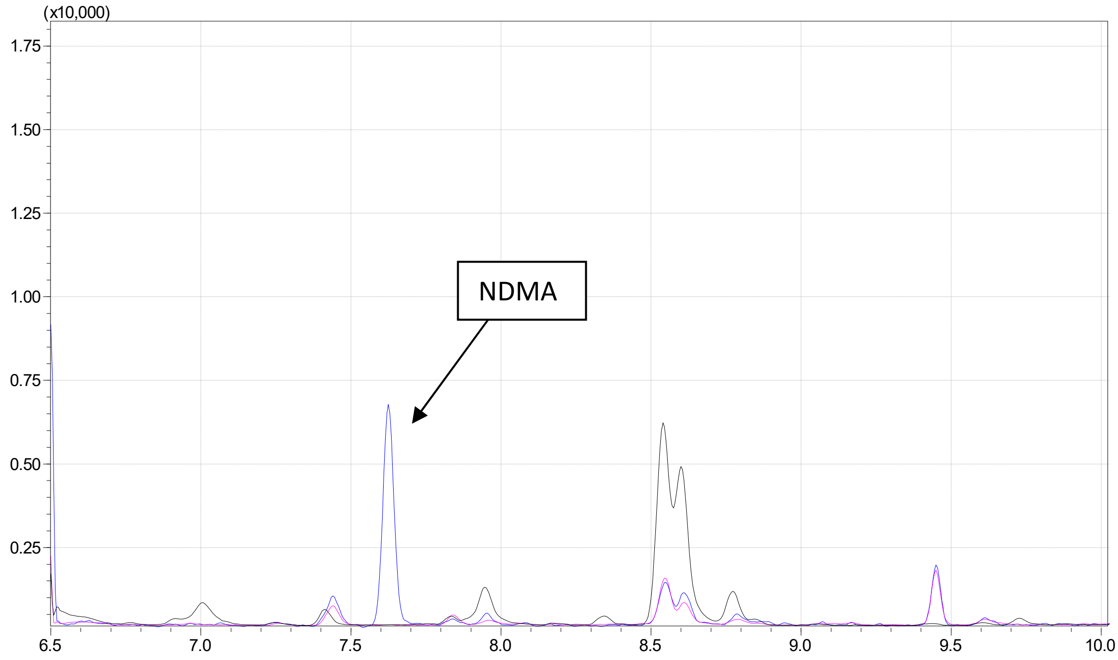

Figure S1. Comparison of chromatograms for NDMA m/z 74 a) blank (black line) b) sample solution of sunitinib (pink line) c) reference sample solution - sunitinib spiked with the standard solution of nitrosamines at concentration level of 18.0 ng/mL (blue line) (CAL 100%).

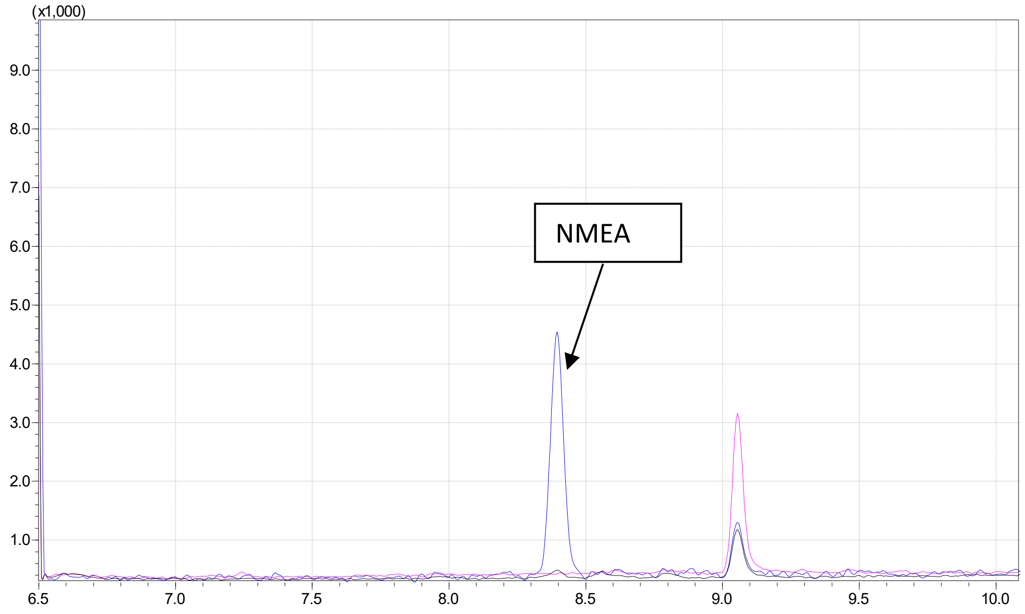

Figure S2. Comparison of chromatograms for NMEA m/z 88 a) blank (black line) b) sample solution of sunitinib (pink line) c) reference sample solution sunitinib spiked with the standard solution of nitrosamines at concentration level of 18.0 ng/mL (blue line) (CAL 100%).

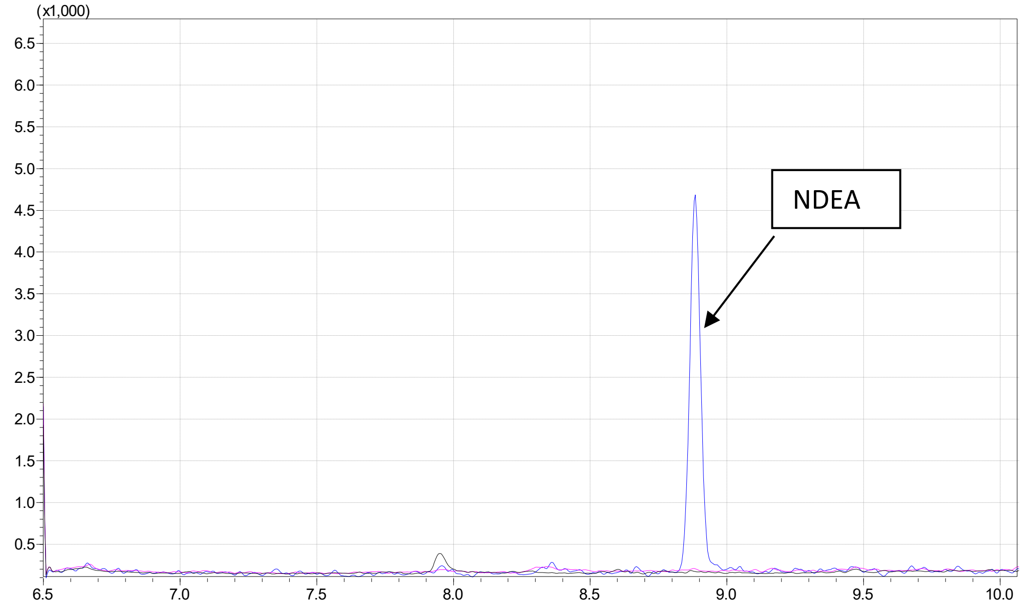

Figure S3. Comparison of chromatograms for NDEA m/z 102 a) blank (black line) b) sample solution of sunitinib (pink line) c) reference sample solution sunitinib spiked with the standard solution of nitrosamines at concentration level of 18.0 ng/mL (blue line) (CAL 100%).

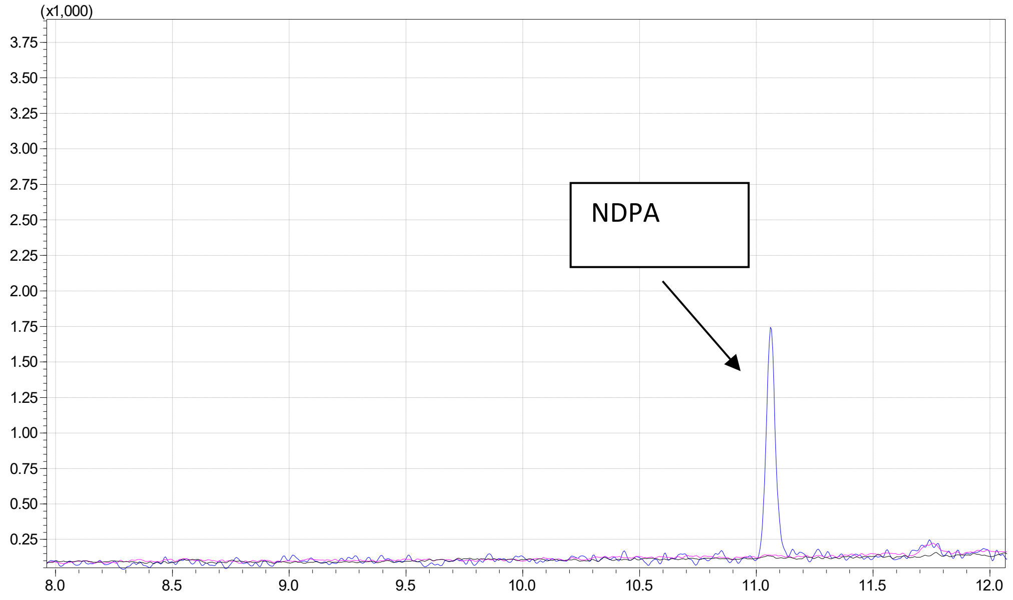

Figure S4. Comparison of chromatograms for NDPA m/z 130 a) blank (black line) b) sample solution of sunitinib (pink line) c) reference sample solution sunitinib spiked with the standard solution of nitrosamines at concentration level of 18.0 ng/mL (blue line) (CAL 100%).

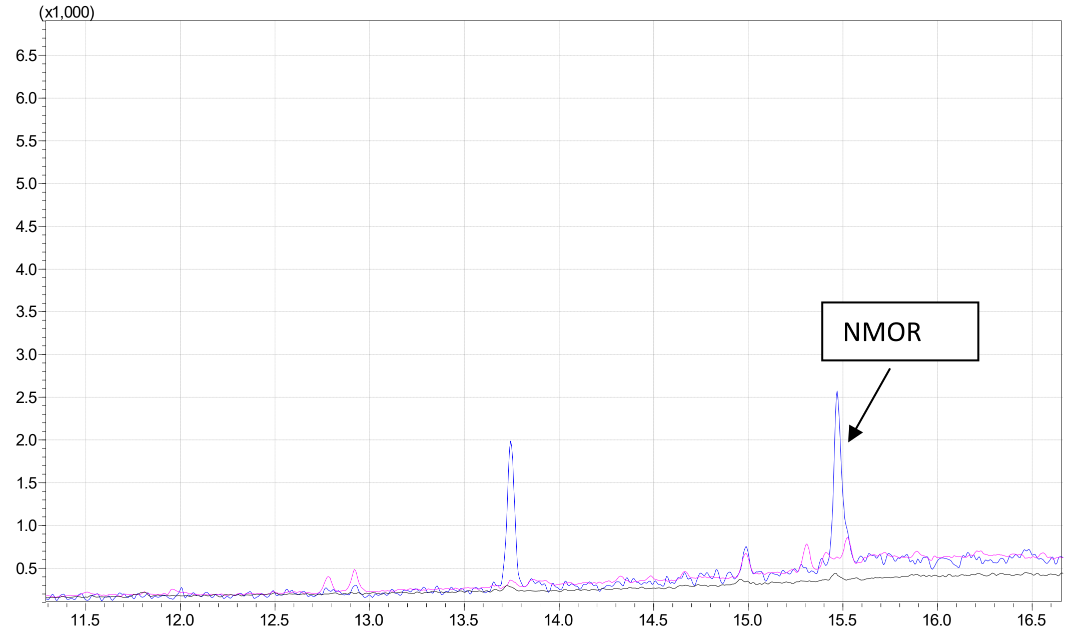

Figure S5. Comparison of chromatograms for NMOR m/z 116 a) blank (black line) b) sample solution of sunitinib (pink line) c) reference sample solution sunitinib spiked with the standard solution of nitrosamines at concentration level of 18.0 ng/mL (blue line) (CAL 100%).

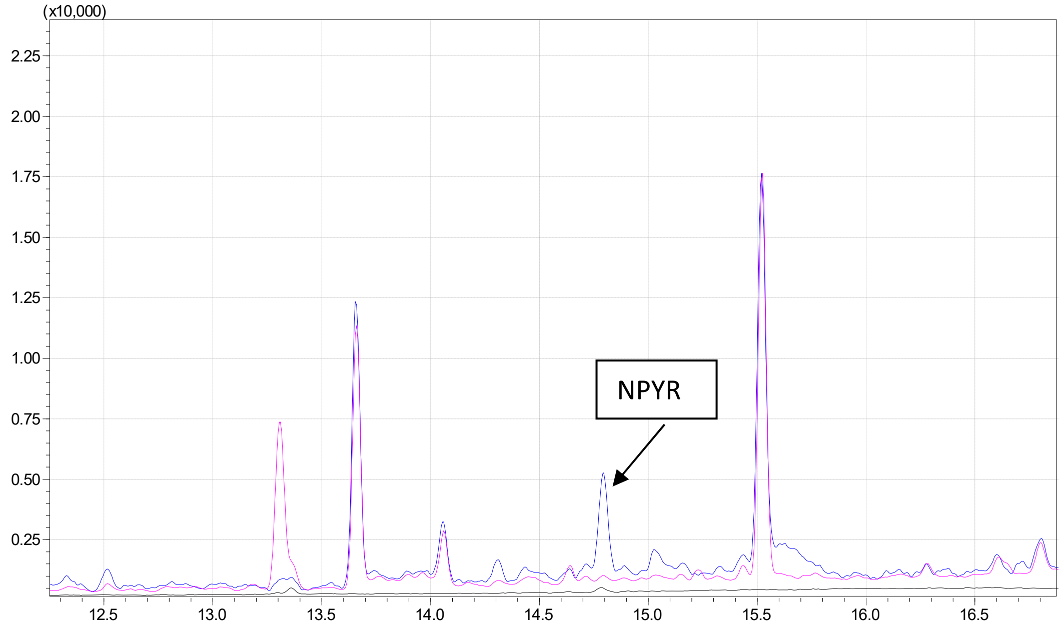

Figure S6. Comparison of chromatograms for NPYR m/z 100 a) blank (black line) b) sample solution of sunitinib (pink line) c) reference sample solution sunitinib spiked with the standard solution of nitrosamines at concentration level of 18.0 ng/mL (blue line) (CAL 100%).

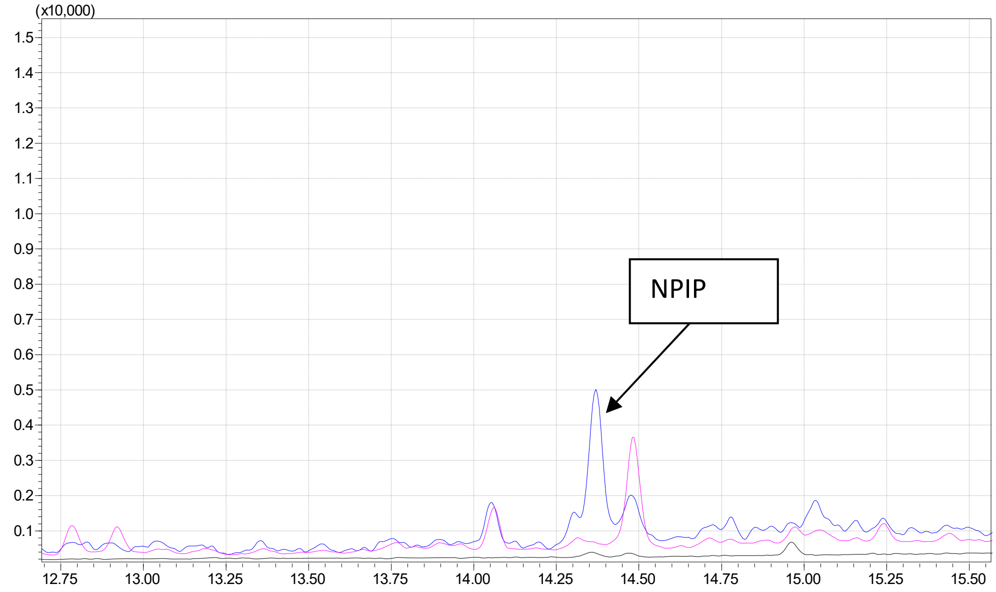

Figure S7. Comparison of chromatograms for NPIP m/z 114 a) blank (black line) b) sample solution of sunitinib (pink line) c) reference sample solution sunitinib spiked with the standard solution of nitrosamines at concentration level of 18.0 ng/mL (blue line) (CAL 100%).

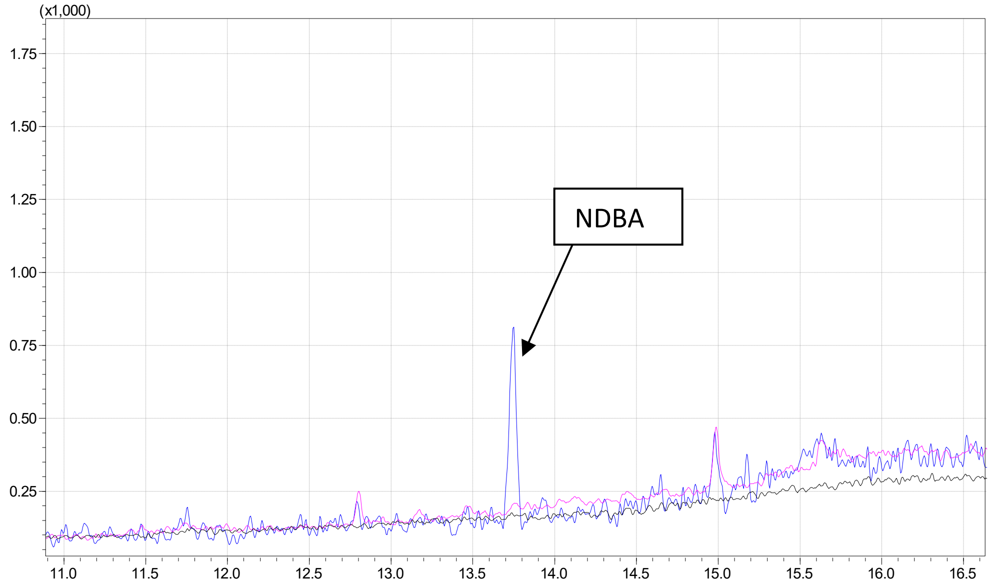

Figure S8. Comparison of chromatograms for NDBA m/z 158 a) blank (black line) b) sample solution of sunitinib (pink line) c) reference sample solution sunitinib spiked with the standard solution of nitrosamines at concentration level of 18.0 ng/mL (blue line) (CAL 100%).

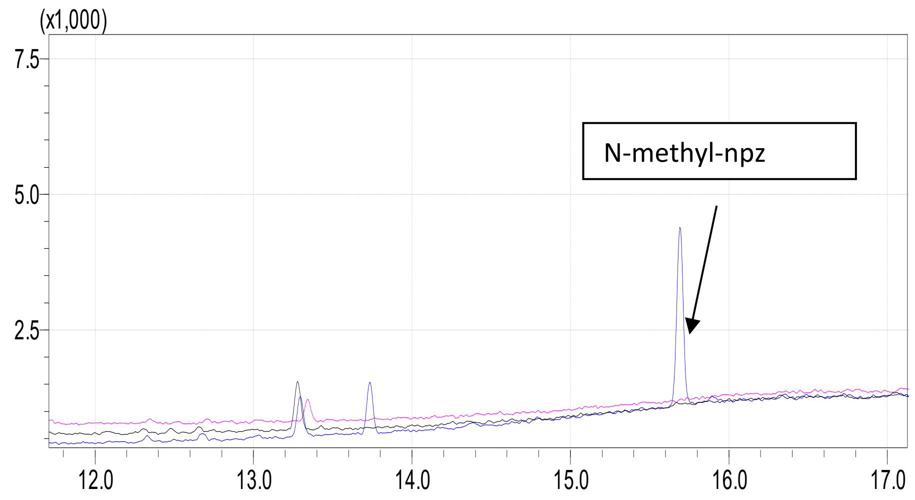

Figure S9. Comparison of chromatograms for N-methyl-npz m/z =99 a) blank (black line) b) sample solution of sunitinib (pink line) c) reference sample solution sunitinib spiked with the standard solution of nitrosamines at concentration level of 18.0 ng/mL (blue line) (CAL 100%).

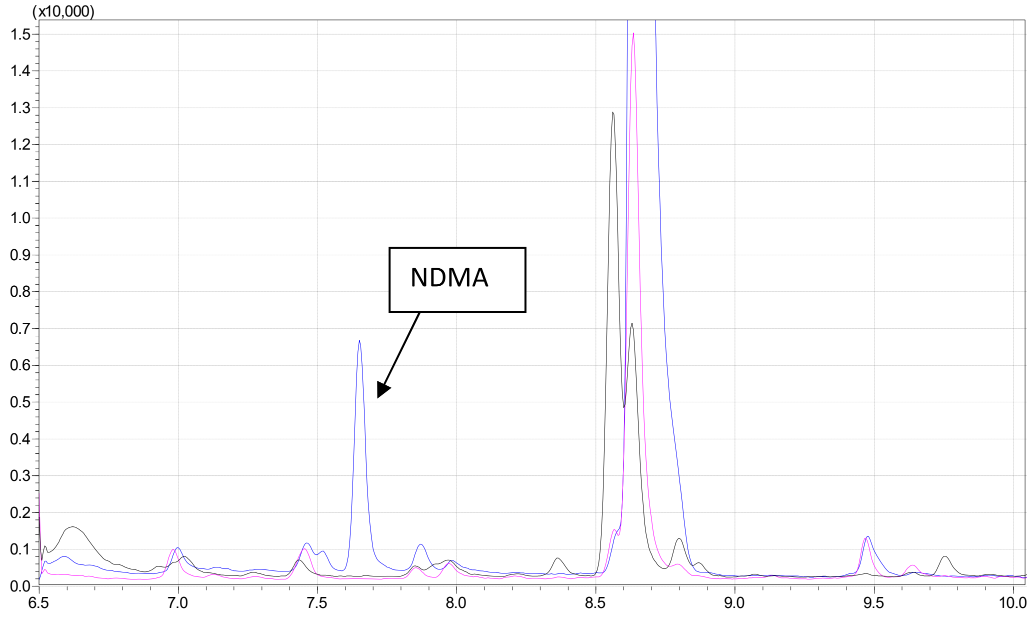

Figure S10. Comparison of chromatograms for NDMA m/z 74 a) blank (black line) b) sample solution of cilostazol (pink line) c) reference sample solution cilostazol spiked with the standard solution of nitrosamines at concentration level of 18.0 ng/mL (blue line) (CAL 100%).

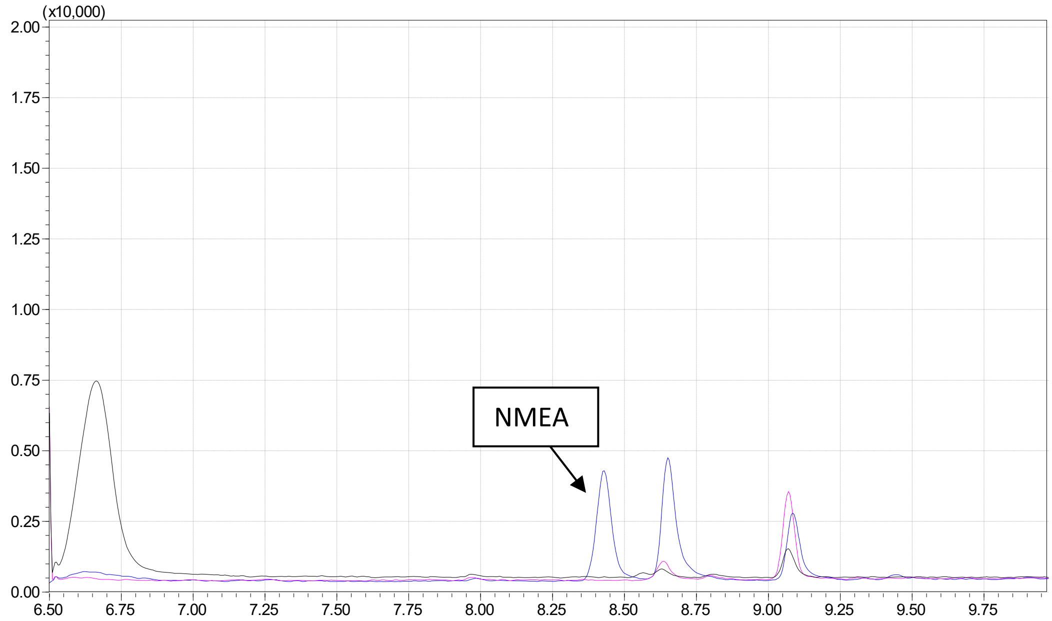

Figure S11. Comparison of chromatograms for NMEA m/z 88 a) blank (black line) b) sample solution of cilostazol (pink line) c) reference sample solution cilostazol spiked with the standard solution of nitrosamines at concentration level of 18.0 ng/mL (blue line) (CAL 100%).

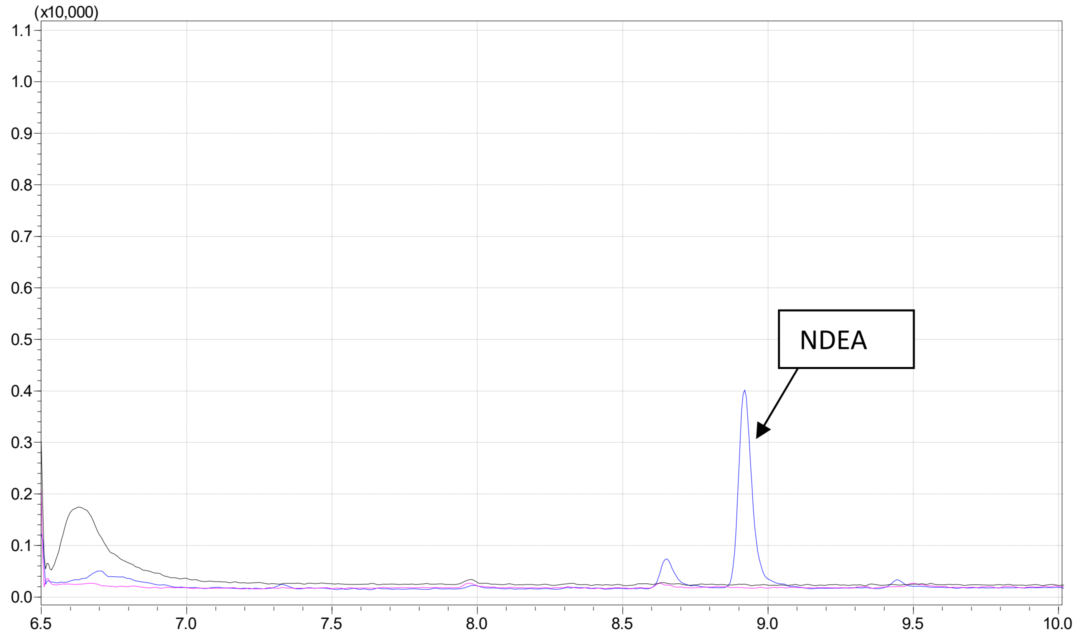

Figure S12. Comparison of chromatograms for NDEA m/z 102 a) blank (black line) b) sample solution of cilostazol (pink line) c) reference sample solution cilostazol spiked with the standard solution of nitrosamines at concentration level of 18.0 ng/mL (blue line) (CAL 100%).

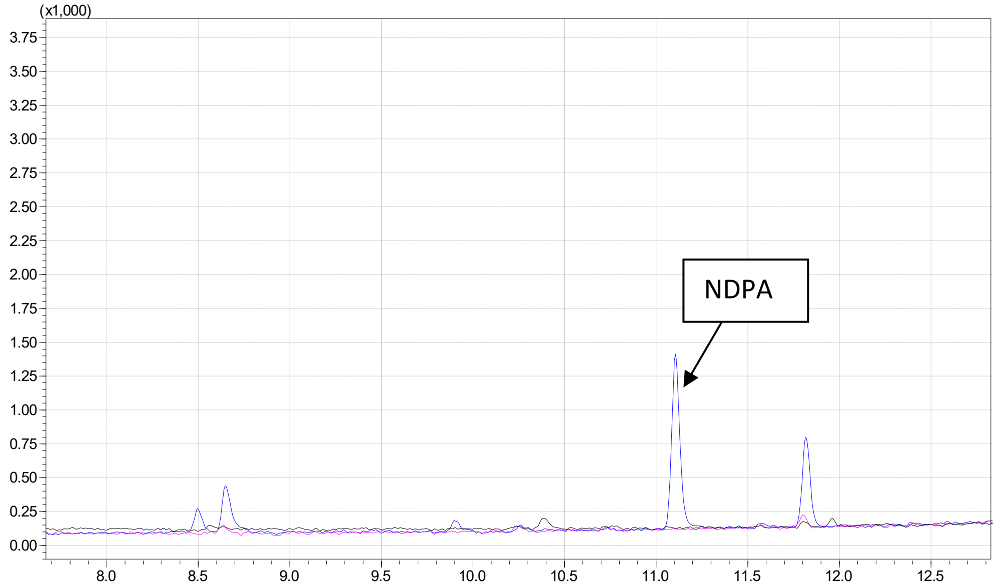

Figure S13. Comparison of chromatograms for NDPA m/z 130 a) blank (black line) b) sample solution of cilostazol (pink line) c) reference sample solution cilostazol spiked with the standard solution of nitrosamines at concentration level of 18.0 ng/mL (blue line) (CAL 100%).

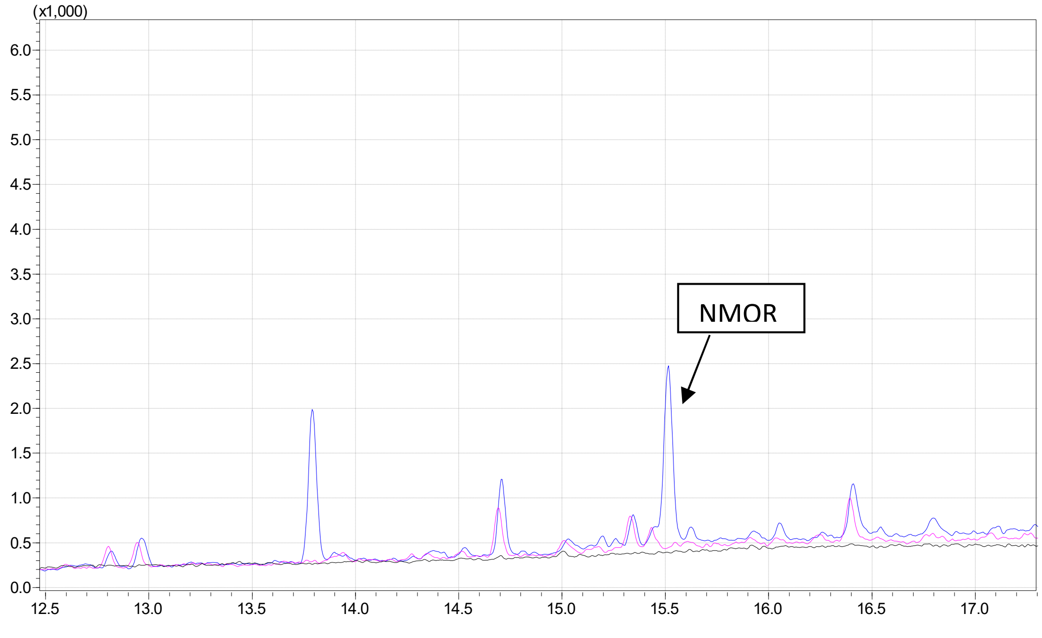

Figure S14. Comparison of chromatograms for NMOR m/z 116 a) blank (black line) b) sample solution of cilostazol (pink line) c) reference sample solution cilostazol spiked with the standard solution of nitrosamines at concentration level of 18.0 ng/mL (blue line) (CAL 100%).

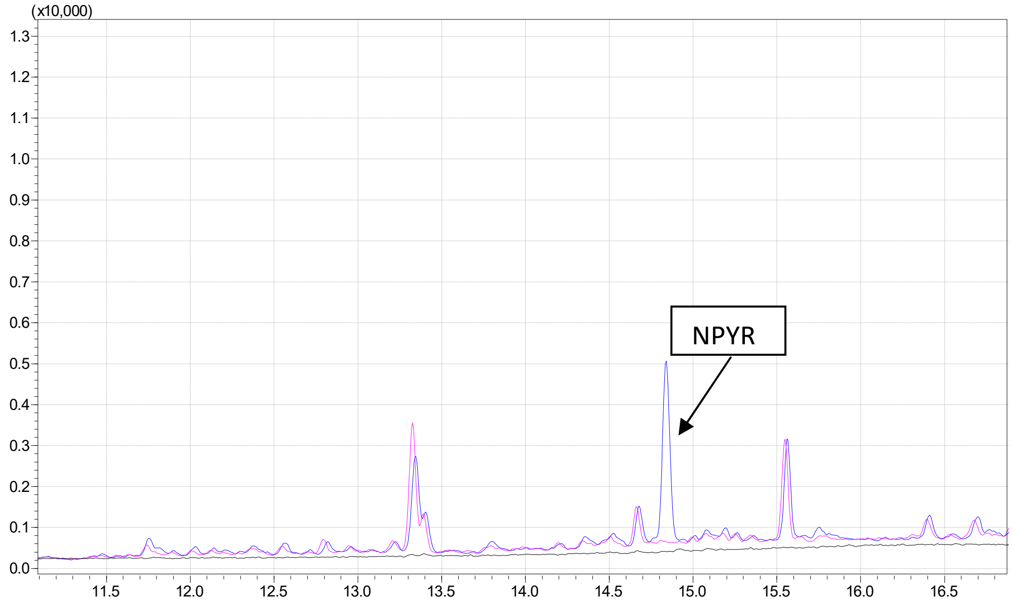

Figure S15. Comparison of chromatograms for NPYR m/z 100 a) blank (black line) b) sample solution of cilostazol (pink line) c) reference sample solution cilostazol spiked with the standard solution of nitrosamines at concentration level of 18.0 ng/mL (blue line) (CAL 100%).

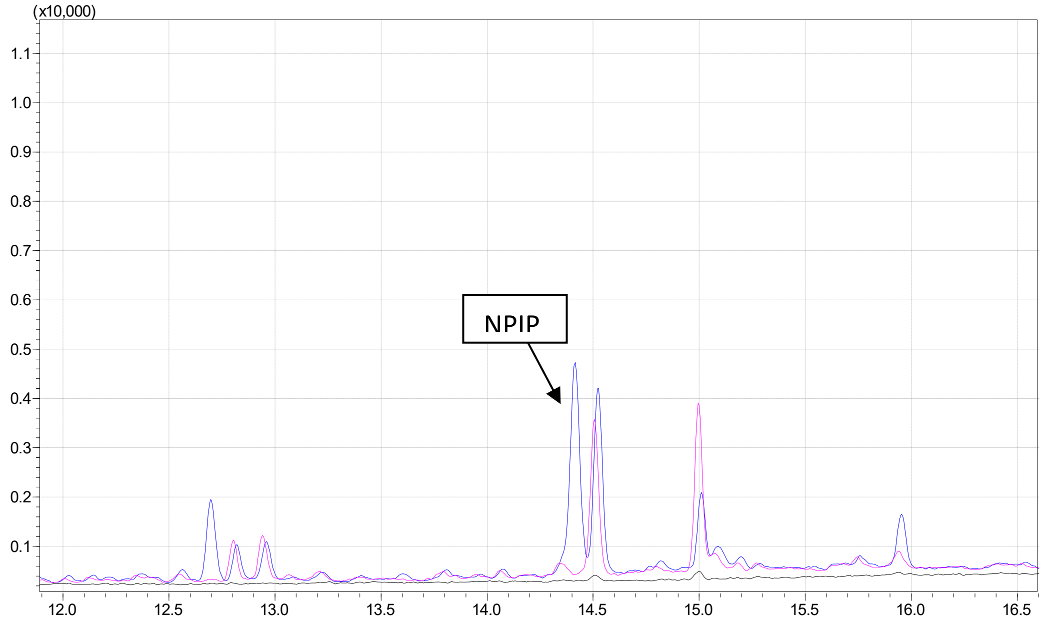

Figure S16. Comparison of chromatograms for NPIP m/z 114 a) blank (black line) b) sample solution of cilostazol (pink line) c) reference sample solution cilostazol spiked with the standard solution of nitrosamines at concentration level of 18.0 ng/mL (blue line) (CAL 100%).

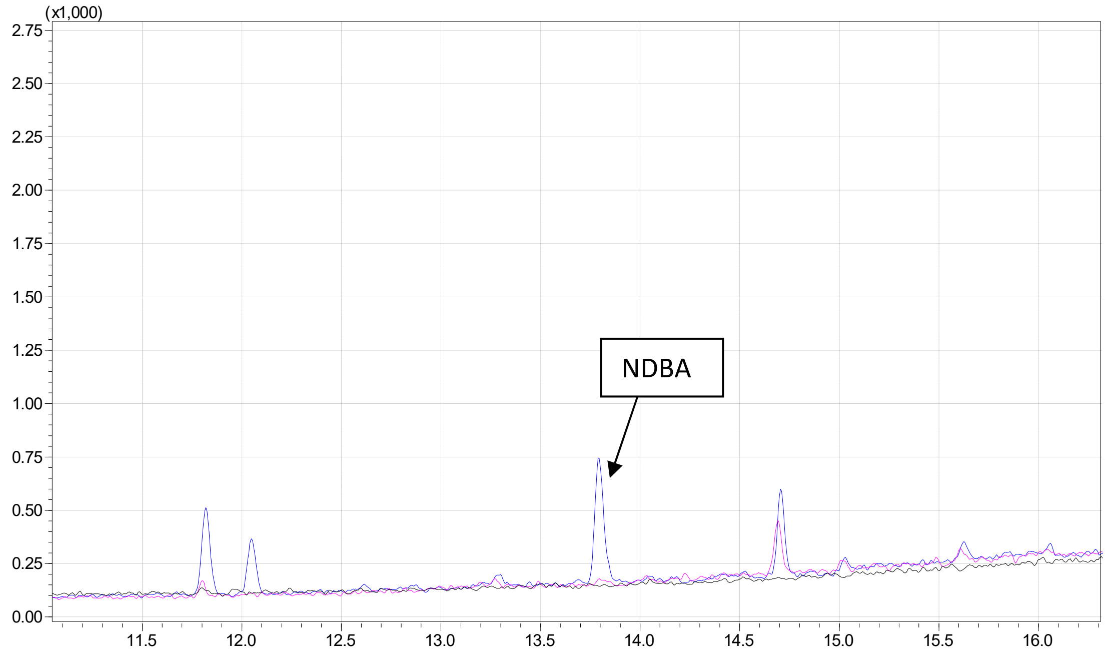

Figure S17. Comparison of chromatograms for NDBA m/z 158 a) blank (black line) b) sample solution of cilostazol (pink line) c) reference sample solution cilostazol spiked with the standard solution of nitrosamines at concentration level of 18.0 ng/mL (blue line) (CAL 100%).

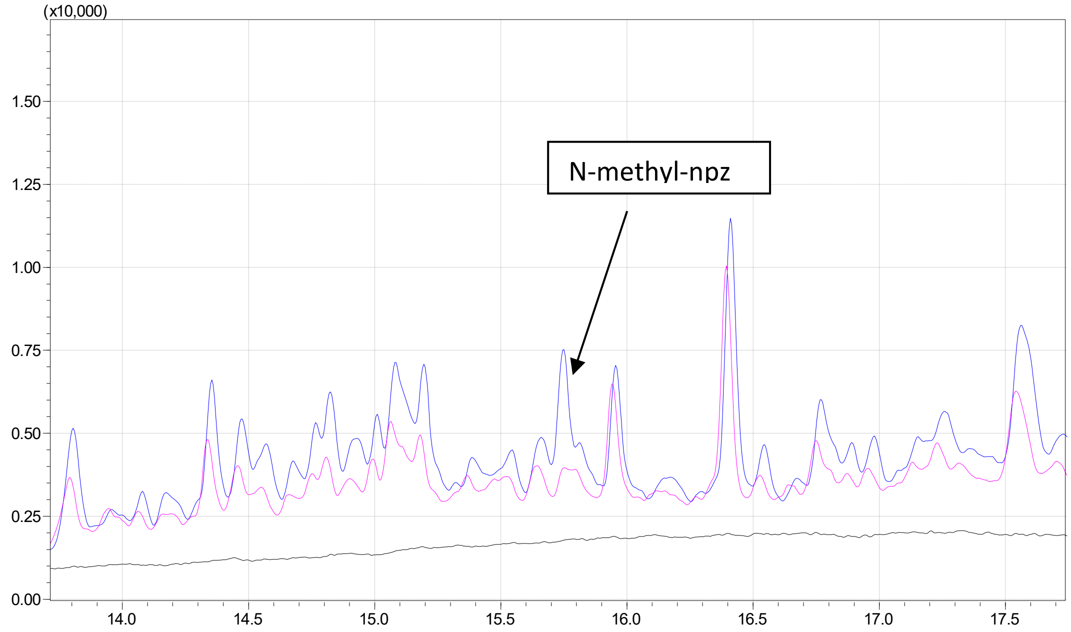

Figure S18. Comparison of chromatograms for N-methyl-npz m/z 99 a) blank (black line) b) sample solution of cilostazol (pink line) c) reference sample solution cilostazol spiked with the standard solution of nitrosamines at concentration level of 18.0 ng/mL (blue line) (CAL 100%).

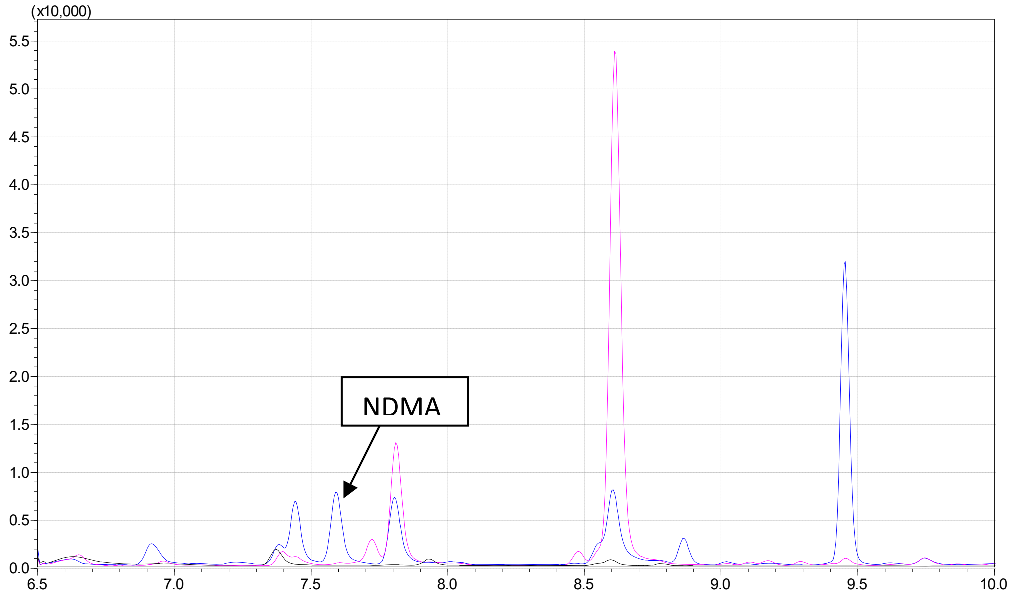

Figure S19. Comparison of chromatograms for NDMA m/z 74 a) blank (black line) b) sample solution of Olmesartan medoxomil (pink line) c) reference sample solution Olmesartan medoxomil spiked with the standard solution of nitrosamines at concentration level of 18.0 ng/mL (blue line) (CAL 100%)).

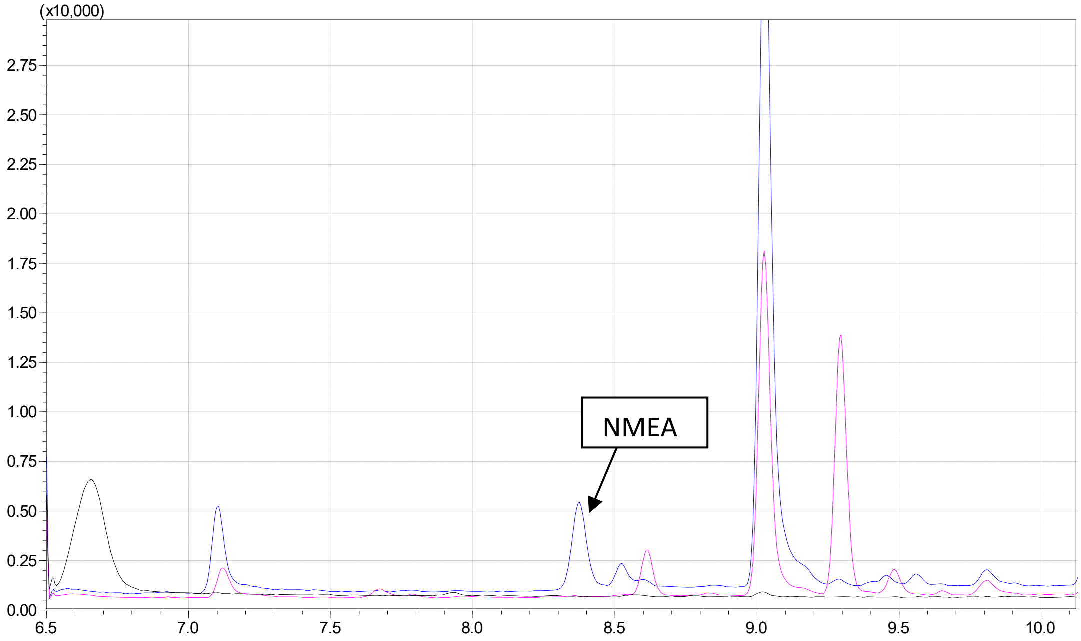

Figure S20. Comparison of chromatograms for NMEA m/z 88 a) blank (black line) b) sample solution of Olmesartan medoxomil (pink line) c) reference sample solution Olmesartan medoxomil spiked with the standard solution of nitrosamines at concentration level of 18.0 ng/mL (blue line) (CAL 100%)).

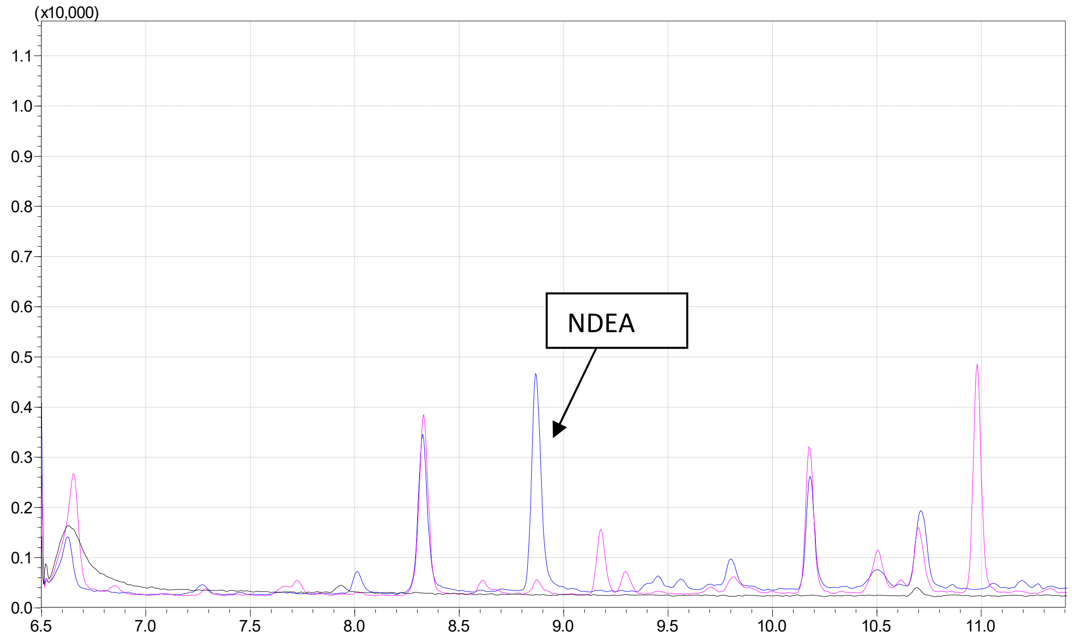

Figure S21. Comparison of chromatograms for NDEA m/z 102 a) blank (black line) b) sample solution of Olmesartan medoxomil (pink line) c) reference sample solution Olmesartan medoxomil spiked with the standard solution of nitrosamines at concentration level of 18. 0 ng/mL (blue line) (CAL 100%)).

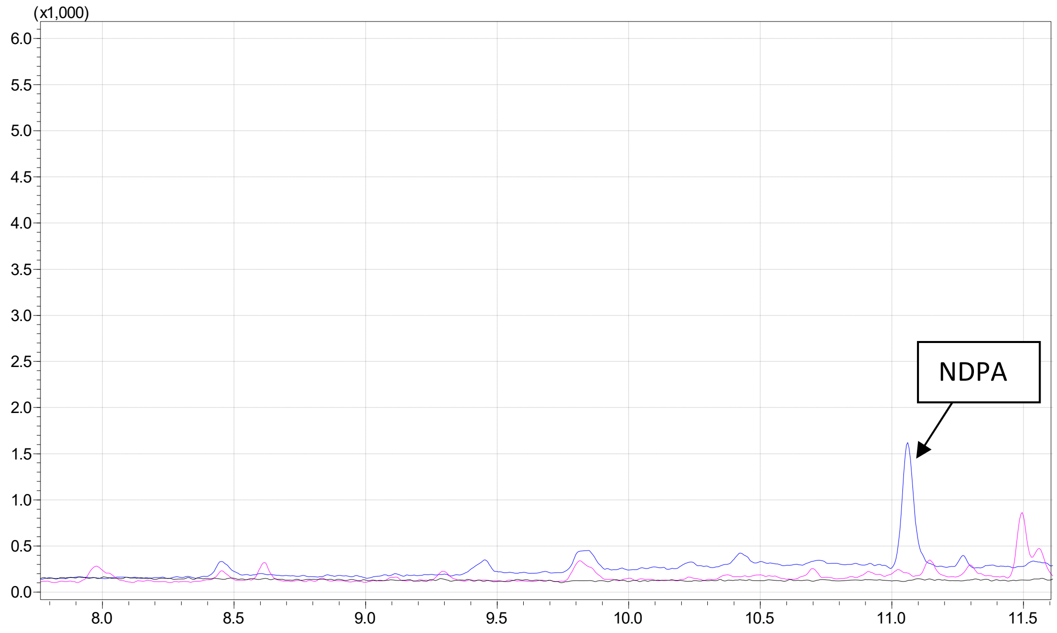

Figure S22. Comparison of chromatograms for NDPA m/z 130 a) blank (black line) b) sample solution of Olmesartan medoxomil (pink line) c) reference sample solution Olmesartan medoxomil spiked with the standard solution of nitrosamines at concentration level of 18. 0 ng/mL (blue line) (CAL 100%)).

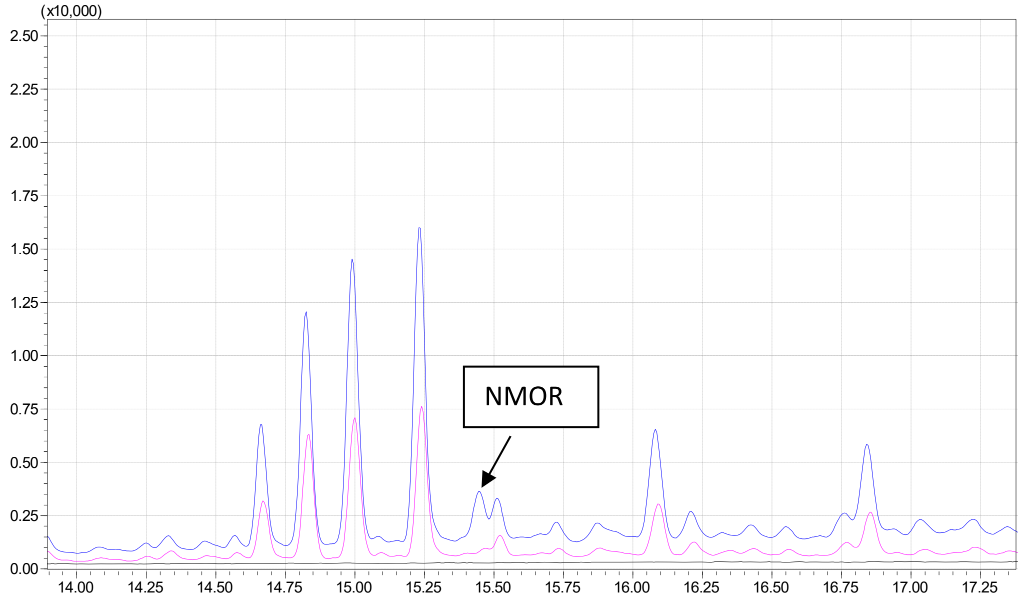

Figure S23. Comparison of chromatograms for NMOR m/z 116 a) blank (black line) b) sample solution of Olmesartan medoxomil (pink line) c) reference sample solution Olmesartan medoxomil spiked with the standard solution of nitrosamines at concentration level of 18. 0 ng/mL (blue line) (CAL 100%)).

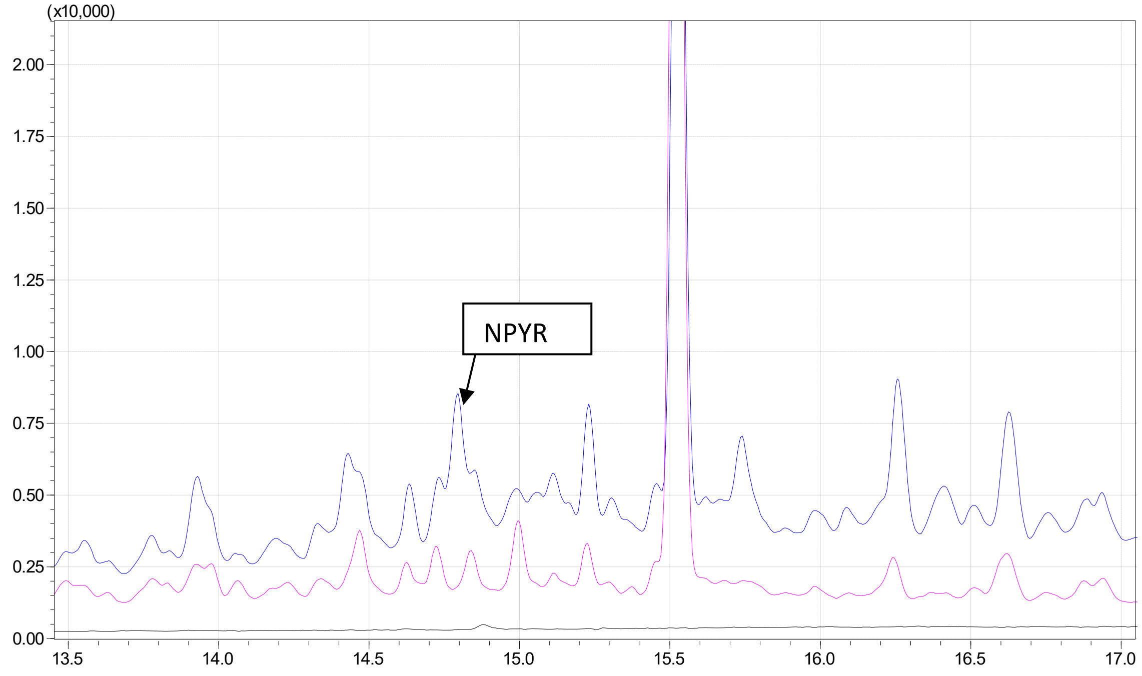

Figure S24. Comparison of chromatograms for NPYR m/z 100 a) blank (black line) b) sample solution of Olmesartan medoxomil (pink line) c) reference sample solution Olmesartan medoxomil spiked with the standard solution of nitrosamines at concentration level of 18. 0 ng/mL (blue line) (CAL 100%)).

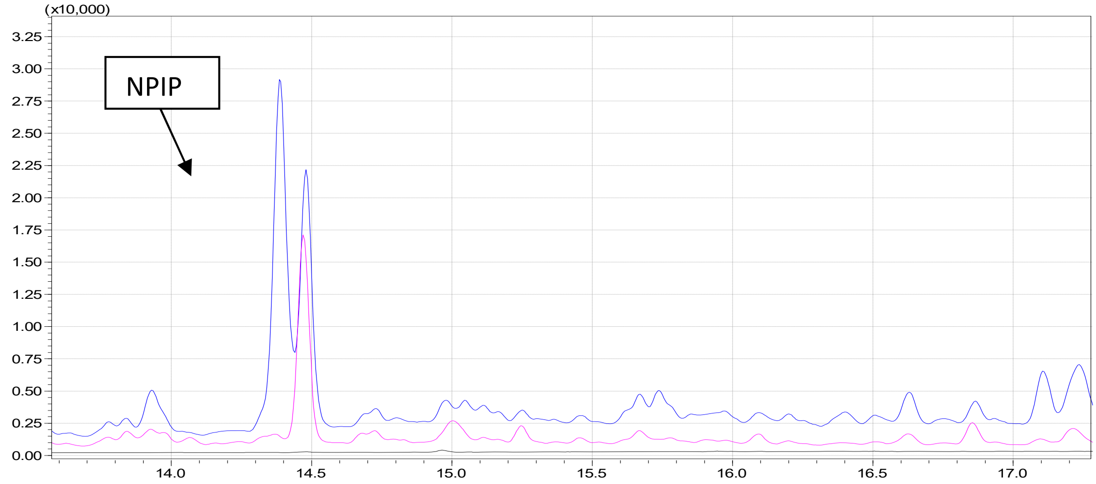

Figure S25. Comparison of chromatograms for NPIP m/z 114 a) blank (black line) b) sample solution of Olmesartan medoxomil (pink line) c) reference sample solution Olmesartan medoxomil spiked with the standard solution of nitrosamines at concentration level of 18. 0 ng/mL (blue line) (CAL 100%)).

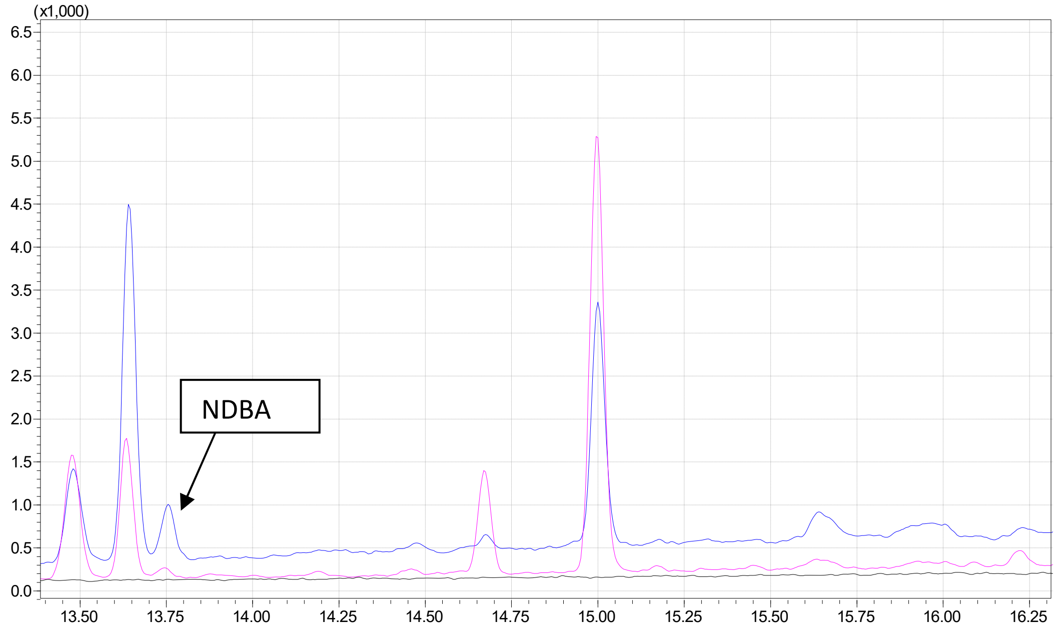

Figure S26. Comparison of chromatograms for NDBA m/z 158 a) blank (black line) b) sample solution of Olmesartan medoxomil (pink line) c) reference sample solution Olmesartan medoxomil spiked with the standard solution of nitrosamines at concentration level of 18. 0 ng/mL (blue line) (CAL 100%)).

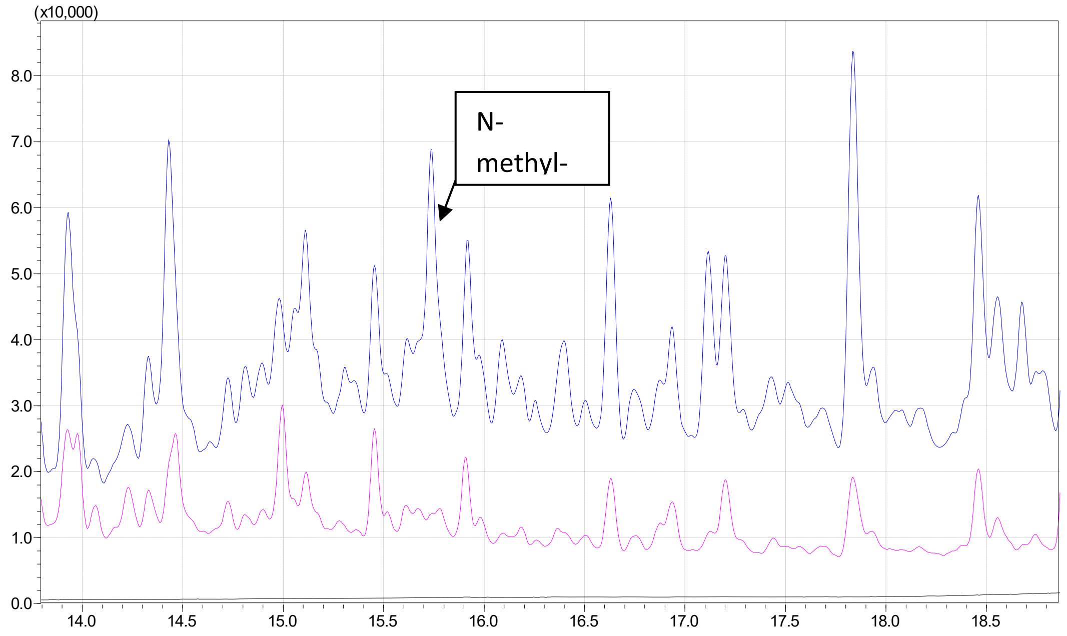

Figure S27. Comparison of chromatograms for N-methyl-npz m/z99 a) blank (black line) b) sample solution of Olmesartan medoxomil (pink line) c) reference sample solution Olmesartan medoxomil spiked with the standard solution of nitrosamines at concentration level of 18. 0 ng/mL (blue line) (CAL 100%)).

Supplementary Note S1 –Calculations 1

The recovery (R) in percent is calculated from the following formula:

$$\text{Recovery} = \frac{C_{cal}}{C} \cdot 100\%$$

where:

C - real concentration of solution, %

C_{cal} - calculated concentration of solution, %

Confidence interval (CI) was calculated using the following formula:

$CI = t_{(\alpha\ 0.05.\ n-1)} \cdot SD/\sqrt{n}$

where:

SD – standard deviation.

n – number of data.

t – Student's t-test at the 0.05 percent confidence level with n-1 degrees of freedom

Concentration of solution was calculated using a linear regression from LOQ to 120% of specification limit (y=ax+b).

Standard deviation and relative standard deviation were calculated using the following formulas:

$$SD = \sqrt{\frac{\sum_{i=1}^{n}(y_i - y_{mean})^2}{n-1}}$$

$$RSD = \frac{SD}{y_{mean}} \cdot 100\%$$

where:

y – individual result

y_{mean} – mean of results

n – population of the sample

Confidence interval (CI) was calculated using the following formula:

$$CI = t_{(\alpha\ 0.05.\ n-1)} \cdot SD/\sqrt{n}$$

where:

SD – standard deviation.

n – number of data.

t – Student's t-test at the 0.05 percent confidence level with n-1 degrees of freedom

Horwitz`s test was calculated using the following formulas:

$$HORRAT(r) = RSD_{Ccal}/PRSD(R)$$

$$PRSD(R) = 2 * C \text{^}(-0.15)$$

where:

HORRAT – the ratio of reproducibility relative standard deviation

$RSDC_{cal}$ – relative standard deviation of the concentration calculated results. %

PRSD(R) – predicted relative standard deviation. %

C –concentration, expressed as a decimal fraction

References:

1. Alshehri, Y.M.; Alghamdi, T.S.; Aldawsari, F.S. HS-SPME-GC-MS as an Alternative Method for NDMA Analysis in Ranitidine Products. *J. Pharm. Biomed. Anal.* **2020**, *191*, 113582, doi:10.1016/j.jpba.2020.113582.

2. Giménez-Campillo, C.; Pastor-Belda, M.; Campillo, N.; Hernández-Córdoba, M.; Viñas, P. Development of a New Methodology for the Determination of N-Nitrosamines Impurities in Ranitidine Pharmaceuticals Using Microextraction and Gas Chromatography-Mass Spectrometry. *Talanta* **2021**, *223*, doi:10.1016/j.talanta.2020.121659.

3. Liu, J.; Xie, B.; Mai, B.; Cai, Q.; He, R.; Guo, D.; Zhang, Z.; Fan, J.; Zhang, W. Development of a Sensitive and Stable GC-MS/MS Method for Simultaneous Determination of Four N-Nitrosamine Genotoxic Impurities in Sartan Substances. *J. Anal. Sci. Technol.* **2021**, *12*, doi:10.1186/s40543-020-00254-2.

4. Lim, H.H.; Oh, Y.S.; Shin, H.S. Determination of N-Nitrosodimethylamine and N-Nitrosomethylethylamine in Drug Substances and Products of Sartans, Metformin and Ranitidine by Precipitation and Solid Phase Extraction and Gas Chromatography–Tandem Mass Spectrometry. *J. Pharm. Biomed. Anal.* **2020**, *189*, 113460, doi:10.1016/j.jpba.2020.113460.

5. Tsutsumi, T.; Akiyama, H.; Demizu, Y.; Uchiyama, N.; Masada, S.; Tsuji, G.; Arai, R.; Abe, Y.; Hakamatsuka, T.; Izutsu, K.; et al. Analysis of an Impurity, N-Nitrosodimethylamine, in Valsartan Drug Substances and Associated Products Using GC-MS. *Biol. Pharm. Bull.* **2019**, *42*, 547–551, doi:10.1248/bpb.b19-00006.

6. FDA Combined Headspace N-Nitrosodimethylamine (NDMA), N-Nitrosodiethylamine (NDEA), N-Nitrosoethylisopropylamine (NEIPA), and N-Nitrosodiisopropylamine (NDIPA) Impurity Assay by GC-MS/MS. **2019**, 1–7.

7. New OMCL Method for Simultaneous Determination of NDMA and NDEA in Sartan. Available online: https://www.edqm.eu/documents/52006/71923/Ad-hoc-projects-OMCL-Network-ranitidine.pdf/f775fbf3-705e-ce82-d1e1-8eb3c06002d4?t=1628667875462.

8. Wichitnithad, W.; Sudtanon, O.; Srisunak, P.; Cheewatanakornkool, K.; Nantaphol, S.; Rojsitthisak, P. Development of a Sensitive Headspace Gas Chromatography–Mass

Spectrometry Method for the Simultaneous Determination of Nitrosamines in Losartan Active Pharmaceutical Ingredients. *ACS Omega* **2021**, *6*, 11048–11058, doi:10.1021/acsomega.1c00982.

9. Lee, D.H.; Hwang, S.H.; Park, S.; Lee, J.; Oh, H. Bin; Han, S.B.; Liu, K.-H.; Lee, Y.-M.; Pyo, H.S.; Hong, J. A Solvent-Free Headspace GC/MS Method for Sensitive Screening of N-Nitrosodimethylamine in Drug Products. *Anal. Methods* **2021**, *13*, 3402–3409, doi:10.1039/D1AY01036K.
